# Social mindfulness predicts concern for nature and immigrants across 36 nations

**DOI:** 10.1038/s41598-022-25538-y

**Published:** 2022-12-21

**Authors:** Kelly Kirkland, Paul A. M. Van Lange, Niels J. Van Doesum, Cesar Acevedo-Triana, Catherine E. Amiot, Liisi Ausmees, Peter Baguma, Oumar Barry, Maja Becker, Michal Bilewicz, Watcharaporn Boonyasiriwat, Thomas Castelain, Giulio Costantini, Girts Dimdins, Agustín Espinosa, Gillian Finchilescu, Ronald Fischer, Malte Friese, Ángel Gómez, Roberto González, Nobuhiko Goto, Peter Halama, Ruby D. Ilustrisimo, Gabriela M. Jiga-Boy, Peter Kuppens, Steve Loughnan, Marijana Markovik, Khairul A. Mastor, Neil McLatchie, Lindsay M. Novak, Ike E. Onyishi, Müjde Peker, Muhammad Rizwan, Mark Schaller, Eunkook M. Suh, William B. Swann, Eddie M. W. Tong, Ana Torres, Rhiannon N. Turner, Christin-Melanie Vauclair, Alexander Vinogradov, Zhechen Wang, Victoria Wai Lan Yeung, Brock Bastian

**Affiliations:** 1 Melbourne School of Psychological Sciences, University of Melbourne, Melbourne, Australia; 2 Department of Experimental and Applied Psychology, Vrije Universiteit Amsterdam, Amsterdam, The Netherlands; 3 Social, Economic and Organisational Psychology, Leiden University, Leiden, The Netherlands; 4 School of Psychology, Universidad Pedagógica y Tecnológica de Colombia, Tunja, Colombia; 5 Department of Psychology, Université du Québec à Montréal, Montreal, Canada; 6 Institute of Psychology, University of Tartu, Tartu, Estonia; 7 Department of Educational, Organizational and Social Psychology, Makerere University, Kampala, Uganda; 8 Department of Psychology, Cheikh Anta Diop University, Dakar, Senegal; 9 CLLE, CNRS, Université de Toulouse, Toulouse, France; 10 Faculty of Psychology, University of Warsaw, Warsaw, Poland; 11 Faculty of Psychology, Chulalongkorn University, Bangkok, Thailand; 12 Serra Húnter Fellow, Department of Psychology, University of Girona, Girona, Spain; 13 Department of Psychology, University of Milan-Bicocca, Milan, Italy; 14 Department of Psychology, University of Latvia, Riga, Latvia; 15 Departamento Académico de Psicología, Pontificia Universidad Católica del Perú, Lima, Peru; 16 Psychology Department, University of the Witwatersrand, Johannesburg, South Africa; 17 School of Psychology, Victoria University of Wellington, Wellington, New Zealand; 18 Department of Psychology, Saarland University, Saarbrücken, Germany; 19 Facultad de Psicología, Universidad Nacional de Educación a Distancia, UNED, Madrid, Spain; 20 Escuela de Psicología, Pontificia Universidad Católica de Chile, Santiago, Chile; 21 Graduate School of Social Sciences, Hitotsubashi University, Kunitachi, Japan; 22 Centre of Social and Psychological Sciences, The Slovak Academy of Sciences, Bratislava, Slovakia; 23 Department of Psychology, University of San Carlos, Cebu City, Philippines; 24 School of Psychology, Swansea University, Swansea, Wales, UK; 25 Faculty of Psychology and Educational Sciences, KU Leuven, Leuven, Belgium; 26 School of Philosophy, Psychology and Language Sciences, The University of Edinburgh, Edinburgh, Scotland, UK; 27 Institute for Sociological Political and Juridical Research, Ss Cyril and Methodius University in Skopje, Skopje, Republic of Macedonia; 28 School of Liberal Studies, Universiti Kebangsaan Malaysia, Bangi, Malaysia; 29 Faculty of Science and Technology, Lancaster University, Lancaster, England, UK; 30 Department of Psychology, University of Illinois Chicago, Chicago, IL USA; 31 School of Psychology, University of Nigeria, Nsukka, Nigeria; 32 Department of Psychology, MEF University, Istanbul, Turkey; 33 Department of Psychology, University of Haripur, Haripur, Pakistan; 34 Department of Psychology, University of British Columbia, Vancouver, Canada; 35 Department of Psychology, Yonsei University, Seoul, South Korea; 36 Psychology Department, The University of Texas, Austin, TX USA; 37 Department of Psychology, National University of Singapore, Singapore, Singapore; 38 Departamento de Psicologia, Federal University of Paraíba, João Pessoa, Brazil; 39 School of Psychology, Queens University Belfast, Belfast, Northern Ireland UK; 40 Department of Social and Organizational Psychology, Instituto Universitário de Lisboa (ISCTE-IUL), CIS-IUL, Lisbon, Portugal; 41 Faculty of Psychology, Taras Shevchenko National University of Kyiv, Kyiv, Ukraine; 42 Department of Psychology, Fudan University, Shanghai, China; 43 Department of Applied Psychology, Lingnan University, Tuen Mun, Hong Kong China

**Keywords:** Psychology, Human behaviour

## Abstract

People cooperate every day in ways that range from largescale contributions that mitigate climate change to simple actions such as leaving another individual with choice – known as social mindfulness. It is not yet clear whether and how these complex and more simple forms of cooperation relate. Prior work has found that countries with individuals who made more socially mindful choices were linked to a higher country environmental performance – a proxy for complex cooperation. Here we replicated this initial finding in 41 samples around the world, demonstrating the robustness of the association between social mindfulness and environmental performance, and substantially built on it to show this relationship extended to a wide range of complex cooperative indices, tied closely to many current societal issues. We found that greater social mindfulness expressed by an individual was related to living in countries with more social capital, more community participation and reduced prejudice towards immigrants. Our findings speak to the symbiotic relationship between simple and more complex forms of cooperation in societies.

Imagine you are the second last person in line at a breakfast buffet, and you’re about to choose a condiment for your toast – there is one peanut butter and two marmalade sachets left. If you choose peanut butter, the stranger behind you will be forced to have marmalade, but if you instead choose marmalade, the person behind you will be left with both options. This is one example of the simple choices people make every day that are mindful to the needs of others – known as social mindfulness (SoMi). While this choice feels relatively inconsequential, it is not yet clear whether such simple and low-cost acts of kindness are related to more complex forms of cooperation, such as better functioning societies or environmental protection. Here we aimed to chart the relationship between simple, context-free acts of cooperation (i.e., SoMi) and more complex forms of cooperation that relate to many of the societal issues we face today.

Social mindfulness (SoMi) entails the decision to leave a hypothetical stranger with choice, and reflects behavior that is sensitive to the needs of others^1^. This form of kindness is encapsulated by the SoMi paradigm which presents individuals with multiple items, where only one item is different and the others are identical. Participants are asked to choose an item knowing that a ‘stranger’ will also get to choose an item after them; the socially mindful option is to pick one of the identical items as this would leave the ‘stranger’ with choice. SoMi has been developed to capture an individual’s inclination to take another’s interests into account when making daily decisions. Importantly, decisions on this task change when participants are asked to make a choice in the absence of another ‘person’^2,3^, discounting alternative explanations such as a preference for more common items. More socially mindful choices have been linked to individual-level factors such as greater empathy, reduced narcissism and increased generosity^4^. However, social mindfulness is a simple and low-cost form of cooperation, that is both context-free and occurs between a hypothetical dyad, and it is unclear whether it relates to more complex forms of cooperation.

The capacity for cooperation is one of the most compelling separations between humans and other species^5,6^. Cooperation can range from decisions that affect the choice of others^1^ to more complex manifestations such as functioning together in larger scale networks or caring for the environment. Here we will focus on two broad forms of complex cooperation that extend beyond context-free, dyadic interactions: a) societal-level trust and positive community participation, and b) inclusive attitudes and moral obligation to protect other people and the environment. First, more complex forms of cooperation often require some degree of certainty that the strangers in one’s society will work together^7,8^. That is, one needs to trust that others will reciprocate and do their part for the betterment of everyone, and this is a critical ingredient in achieving social capital and a sense of cohesion in society. Second, inclusive attitudes towards diverse groups of people likely promotes cooperation with strangers more broadly, rather than just with one’s ingroup. Caring for and protecting the environment is also considered cooperative – combatting climate change is necessary to secure a safe future for humans and all other species^9^.

Few lines of research have explored how and if simple, low-cost cooperative decisions are linked to more complex cooperative outcomes that relate to important societal issues. Yet this association is plausible and likely to be symbiotic. When an accumulation of individuals make socially mindful decisions, this may have a snowballing effect for trust in strangers, more positive societal participation, and a care and concern for the welfare of others more broadly^2^. Second, the opposite direction may be just as likely. Societies that are cooperative – by protecting the environment, promoting concern for all types of people, having positively functioning communities and trust in others – may set a norm where individuals are mindful of the needs of others^10^. Both causal paths are plausible, and the relationship between simple forms of cooperation and more complex forms is likely bidirectional.

To date, one study has found a relationship between SoMi and a complex cooperative outcome in a dataset spanning 31 countries^11^. More socially mindful choices by individuals were associated with a higher Environmental Performance Index (EPI) which rates nations on their positive environmental impact^12^. The authors postulated that SoMi may be a simple way of capturing how individuals treat others more broadly – where we behave with the needs of all people in mind and this in accumulation results in more complex forms of cooperation that strive for the betterment of everyone. However, this was an exploratory finding and confirmatory research is needed. Environmental performance also reflects only one kind of complex cooperative outcome, yet the mechanistic claim is general and should extend to other outcomes. More evidence is therefore needed to confirm the existence of a symbiotic, mutually reinforcing, and generalized relationship between simple and more complex forms of cooperation within a society.

The current study aimed to assess the relationship between SoMi and more complex cooperative outcomes and attitudes that relate to real-world challenges faced by society. To achieve this, we first conducted a preregistered replication to confirm the findings by Van Doesum and colleagues^11^ in a large, multinational dataset with a more diverse sample of countries compared to the prior work. We repeated the analytical approach, including an assessment of the relationship between SoMi and individual-level variables (where available in the current dataset) and several country-level variables. While we conducted a replication of the larger set of results produced by Van Doesum and colleagues^11^ where measures were available, our primary aim was to establish the link between SoMi and the EPI. As pre-registered, we hypothesized that greater SoMi would relate to a higher EPI.

After a successful replication was achieved, we then aimed to explore the relationship between SoMi and a broader range of more complex forms of cooperative outcomes and attitudes. Our goal was not to focus on any specific measure but instead provide broad and comprehensive evidence for our general thesis. We examined the relationship between SoMi and several individual-level and country-level variables that broadly assessed a) indicators of societal-level trust and positive community participation, and b) inclusive attitudes and moral obligation to protect others, including the environment. We hypothesized that more socially mindful choices would be associated with more complex forms of cooperation.

## Method

Ethical approval was obtained by the last author from the Behavioural and Social Sciences Ethical Review Committee, project no. 2009001486. Informed consent was obtained in line with the requirements of ethical approval. This study meets the relevant ethical guidelines for each country involved.

### Participants

The survey was completed by 6665 participants (*M* = 21.59 years, *SD* = 5.72 years) and approximately 63% identified as female. Participants came from 41 universities from 36 countries across the world: Australia, Belgium, Brazil, Canada (English speaking), Canada (French speaking), Chile, China, Colombia, Costa Rica, England, Estonia, France, Germany, Hong Kong, Italy, Japan, Latvia, Macedonia, Malaysia, Netherlands, New Zealand, Nigeria, Northern Ireland, Pakistan, Peru, Philippines, Poland, Portugal, Scotland, Singapore, Slovakia, South Africa, South Korea, Spain, Thailand, Turkey, Uganda, Ukraine, USA (North), USA (South) and Wales. The samples from Canada (French speaking and English speaking), United Kingdom (England, Northern Ireland, Scotland and Wales) and the USA (North and South) were treated as separate locations for the sake of analyses. A minimum of 100 participants completed the survey per location with the exception of Chile (*n* = 69), Estonia (*n* = 63), Thailand (*n* = 99), Wales (*n* = 85). See Supplementary Materials 1 for details on each country.

### Part 1: Preregistered Replication

#### Design

We mirrored the approach by Van Doesum and colleagues^11^ and aimed to replicate the cross-national findings in a new and more culturally diverse sample. For this study, we were primarily interested in confirming the relationship between SoMi and EPI. Participants filled out a questionnaire online or via hardcopy, which had previously been translated from English into the native language of the country. All participants provided informed consent. The data was collected between 2018 and 2019. The individual-level variables used in this experiment were taken from a larger questionnaire that contained other variables not reported here. The country-level measures were collected from a variety of online databases and the information for each is detailed below.

#### Measures

SoMi was measured by presenting participants with 12 questions asking them to choose an item from a series of three or four items^1^. Participants were told that an unknown, hypothetical person will get to make a choice after them for each question. The items varied per question, and included common objects such as pens, clocks, and cupcakes. Critically, all items except one were identical; for example, participants would need to choose between two red mugs and one blue mug. A choice was considered socially mindful when a participant chose the common item instead of the single item, as this left the second ‘person’ with choice. A final SoMi score was obtained by calculating a percentage of the number of socially mindful choices a participant made, and this ranged from 0% (least socially mindful) to 100% (most socially mindful). An additional sample, Senegal, received altered instructions by mistake. Participants selected both the item they would choose as well as the item the other person would receive. We have chosen to remove Senegal (*n* = 778) from the sample to ensure consistency across countries. See Supplementary Materials 2 for the exact question given to participants.

A number of other individual-level measures were taken from the questionnaire (see Supplementary Materials 3 for these items). We measured age, the MacArthur Scale of Subjective Socioeconomic Status^13^ and gender. We further assessed generalized trust using a 6-item scale measuring the trust one has in others and the trust one perceives others to have in them^14^. Trust one has in others was measured with three items, such as ‘I dare to put my fate in the hands of most other people’ (α = 0.59). Trust one perceives others have in them was measured with three items, such as ‘I think that most other people trust me’ (α = 0.53). We further included measures of economic and social conservatism. These measures were not included in the original Van Doesum and colleagues^11^ study but were included here as political preference is frequently related to other forms of prosocial behavior^15^. Several other measures were used in the previous work^11^ but were not available in our current dataset, including Social Value Orientation, participant education, parental education, income, and number of brothers and sisters.

We also included several country-level measures from various online databases (see Supplementary Materials 4 for details). Measures of trust, religiosity and civic cooperation were obtained from the World Values Survey^16^ and European Values Survey^17^. We further used indices for Rule of Law^18^, Democracy^19^, Competitiveness^20^, Press Freedom^21^ and the EPI^12^. We also assessed Hofstede’s cultural dimensions (power distance, individualism, masculinity, uncertainty avoidance, long-term orientation and indulgence)^22^. Finally, we obtained the Gross National Income (GNI) per capita^23^ and Gross Domestic Product (GDP) per capita^24^, as well as the Gini Index^25^.

#### Analytical strategy

Following Van Doesum and colleagues^11^ we analyzed several Linear Mixed Models (LMM) with SoMi as the outcome variable and sample location as the random intercept. This was achieved using the lme4 package in R^26^. First, we calculated a within-countries (country mean centered) and between-countries (grand mean centered for country averages) estimate for each individual-level predictor variable and analysed the effect of these two estimates together in an LMM for each predictor separately. Further, using the country-level measures, we replicated the bivariate relationships that were analyzed in the prior work. Using the country-level variables as fixed effects (grand mean centered), we analyzed the bivariate relationship for each predictor separately and this broadly fell into three categories: (1) key variables, (2) Hofstede’s dimensions and 3) economic indices. For all analyses, our dependent variable, SoMi, was standardized whereby the beta values can be interpreted in a similar way to a Pearson’s coefficient (see approach by Van Doesum and colleagues^11^).

### Part 2: Exploratory examination of link between SoMi and complex cooperation

#### Measures

After replicating the work by Van Doesum and colleagues^11^, we explored the relationship between SoMi and more complex forms of cooperative outcomes and attitudes. We measured a number of individual- and country-level variables that broadly reflected two categories: (1) societal-level trust and positive community participation, and (2) inclusive attitudes and moral obligation to protect others, including the environment. SoMi may relate to these factors because societies where many individuals are mindful of the needs of others may result in a more united community, where individuals have broader care and concern for the needs of people more broadly. The opposite direction is also likely true, whereby highly cooperative, and other-focussed societies may promote a norm that influences individuals to be mindful of the needs of others. The purpose of including each of these broad constructs was to find comprehensive evidence for our thesis as opposed to zooming in on any specific construct. The exact wording for the individual-level measures can be seen in Supplementary Materials 5, and the country-level measures were obtained from a variety of online databases (see Supplementary Materials 6).

First, we assessed several individual-level and country-level measures that broadly relate to societal-level trust and positive community participation, both indicators of more complex cooperation. Perceptions of anomie was included to assess views that the social fabric and leadership of society is breaking down. As communities under a state of anomie are typically characterized by low trust and a perception that everyone is self-interested rather than cooperative, we predicted this would be negatively associated with SoMi. The measure of anomie (α = .83) was comprised of two subscales^27,28^. Anomie in the social fabric is a six-item scale that reflects perceptions of low trust among citizens and no shared moral standards e.g., “People think that there are no clear moral standards to follow” (α = .77). Anomie in leadership was ascertained with six items and referred to perceptions the government is ineffective and illegitimate e.g., “Politicians don’t care about the problems of average person” (α = .81). Further, we assessed how much individuals value benevolence (i.e., by being “loyal, honest, helpful, responsible, and forgiving”) with the with the Schwartz Benevolence value^29^, as valuing this quality represents an individual who is other-oriented.

On the country-level, we additionally obtained measures including trust in others^30^ – a key ingredient in fostering cooperation with strangers – as well as the Social Capital Index^31^ – an indicator of social cohesion between citizens on a national scale. We additionally measured positive global citizenship^32^ and voice and accountability of people^33^ as indicators of how well a country protects the rights, needs and freedom of all its citizens, which paves the way for positive community participation. Finally, measures were obtained from the World Values Survey^16^ and European Values Study^17^ indicating engagement in collective action – an indicator of cooperative community participation – and how much ‘unselfishness’ is valued as a child quality – a measure for how much an individual values doing things for others rather than the self. In line with our reasoning, we predicted that these measures of country-level trust and positive community participation would be positively associated with a simple, dyadic and context-free form of cooperation as measured by SoMi.

Second, we gathered a number of individual-level and country-level measures that represent inclusive attitudes and moral obligation to protect others, including the environment. On the individual-level, the Moral Expansiveness Scale (MES) was used to assess the number of entities one does and does not have moral concern for as a broad indicator for inclusive attitudes towards others^34,35^. Participants were asked to rate their moral concern for entities such as family, outgroup members, animals and nature, and more entities included in one’s ‘moral circle’^36^ indicated greater moral expansiveness (α = .92). We additionally isolated the ‘nature’ entities from the Moral Expansiveness Scale (α = .91), as a more specific indicator of the moral concern individuals have for the environment. Further, as an assessment of inclusive attitudes towards outgroups, we measured attitudes towards immigrants with the average response to a six-item scale, including questions such as “Immigrants abuse the system of social benefits” (α = .94)^37^. Moreover, we assessed how much individuals value “equality, a world at peace, wisdom, social justice, broadmindedness, to enjoy the beauty of nature and the arts, to feel unity with nature and to protect the environment” with the Schwartz Universalism value^29^, as a measure of both care and concern for others more broadly as well as care for the environment. In line with our reasoning that SoMi towards specific others should be related to a broader and more general orientation to inclusion and consideration, we predicted SoMi would be related to more inclusive moral concern and attitudes.

On the country-level, a measure of Environmental Care was obtained to assess how much a country cares and protects the environment^32^. We further obtained measures of tolerance of having minorities as neighbors – as an indicator of care for minority and outgroup individuals – and how important individuals believe it is to teach their children ‘tolerance and respect’ – as a measure of how much individuals value preserving the dignity of others more broadly – from the World Values Survey^16^ and European Values Study^17^.” Again, viewing these as indicators of broader and more general indicators of inclusion and consideration, we predicted these measured would be positively related to SoMi.

We also included four additional demographic variables in each of our models as controls: age, gender, economic conservativism, and social conservativism, measured in an identical manner as described in Part 1.

#### Analytical strategy

We analyzed several Linear Mixed Models with SoMi as the outcome variable and country as the random intercept. This was achieved using the lme4 package in R^26^. Each predictor variable was included in its own model as a fixed effect alongside the four demographic control variables. All individual-level variables were partitioned into a within-countries (country mean centered) and between-countries (grand mean centered for country averages) estimate, and country-level variables were grand mean centered. For all analyses, our dependent variable, SoMi, was standardized whereby the beta values can be interpreted in a similar way to a Pearson’s correlation (see approach by Van Doesum and colleagues^11^).

## Results

### Part 1: Preregistered replication

**Figure 1:**
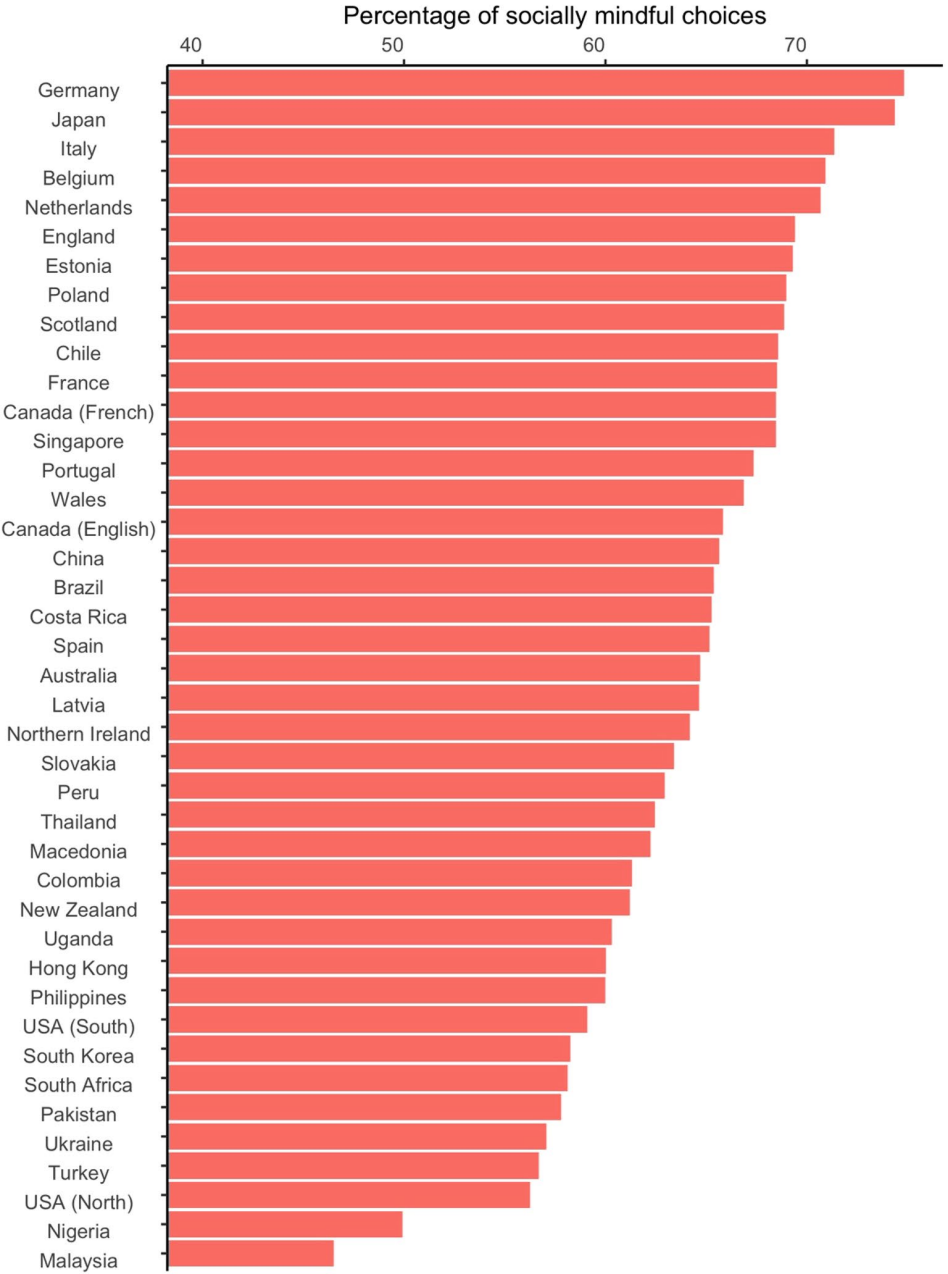
Average percentage of socially mindful choices per country.

Across all countries, we found 63.2% (*SD* = 22.6) of participants’ choices were socially mindful on average. We examined the Intra-Class Correlation (ICC) to establish the amount of variance that can be explained by country in SoMi scores. Approximately 6.6% of the variance in SoMi can be explained by differences between the 41 samples. See Fig. 1 for the average SoMi score per sample. We ran several tests to establish whether the differences between countries were meaningful. A Likelihood Ratio Test found the variance between countries to be greater than zero, χ^2^(40) = 487.39, *p* < .001. Likewise, an Ordinary Least Squares ANOVA found a significant effect of country on the percentage of socially mindful choices, *F*(40, 6149) = 12.59, *p* < .001.

See Supplementary Materials 7 for the full results of each model reported below. Following the approach by Van Doesum and colleagues^11^ we then analyzed a number of individual-level variables collected from participants to establish their relationship with SoMi using seven Linear Mixed Models, with sample location as the random intercept. Here, each variable was partitioned into a within-countries (country mean centered) and between-countries (grand mean centered for country averages) estimate, with both estimates included in the same model. As demonstrated in Table 1, economic liberalism, social liberalism, generalized trust in others and perceptions that others trust you were associated with more socially mindful choices within-countries. Between-countries, the presence of more females as well as greater economic and social liberalism was associated with increased SoMi.

**Table 1:** Replication results for variables recorded at the individual level.

Variables	Within-countries			Between-countries		
	β	*SE*	*p*	β	*SE*	*p*
Age	0.01	0.01	.295	0.12	0.16	.460
Subjective social status	< 0.01	0.01	.773	0.17	0.16	.283
Gender	− 0.01	0.01	.543	0.61	0.14	< .001***
Economic conservatism	− 0.05	0.01	< .001***	− 0.50	0.14	<.001***
Social conservatism	− 0.03	0.01	.010*	− 0.63	0.12	< .001***
Trust in others	0.05	0.01	<.001***	0.20	0.16	.210
Trust towards self	0.03	0.01	.037*	0.19	0.16	.244

Gender was coded as male (1) and female (2).

*p < .05, **p < .01, ***p < .001.

**Figure 2:**
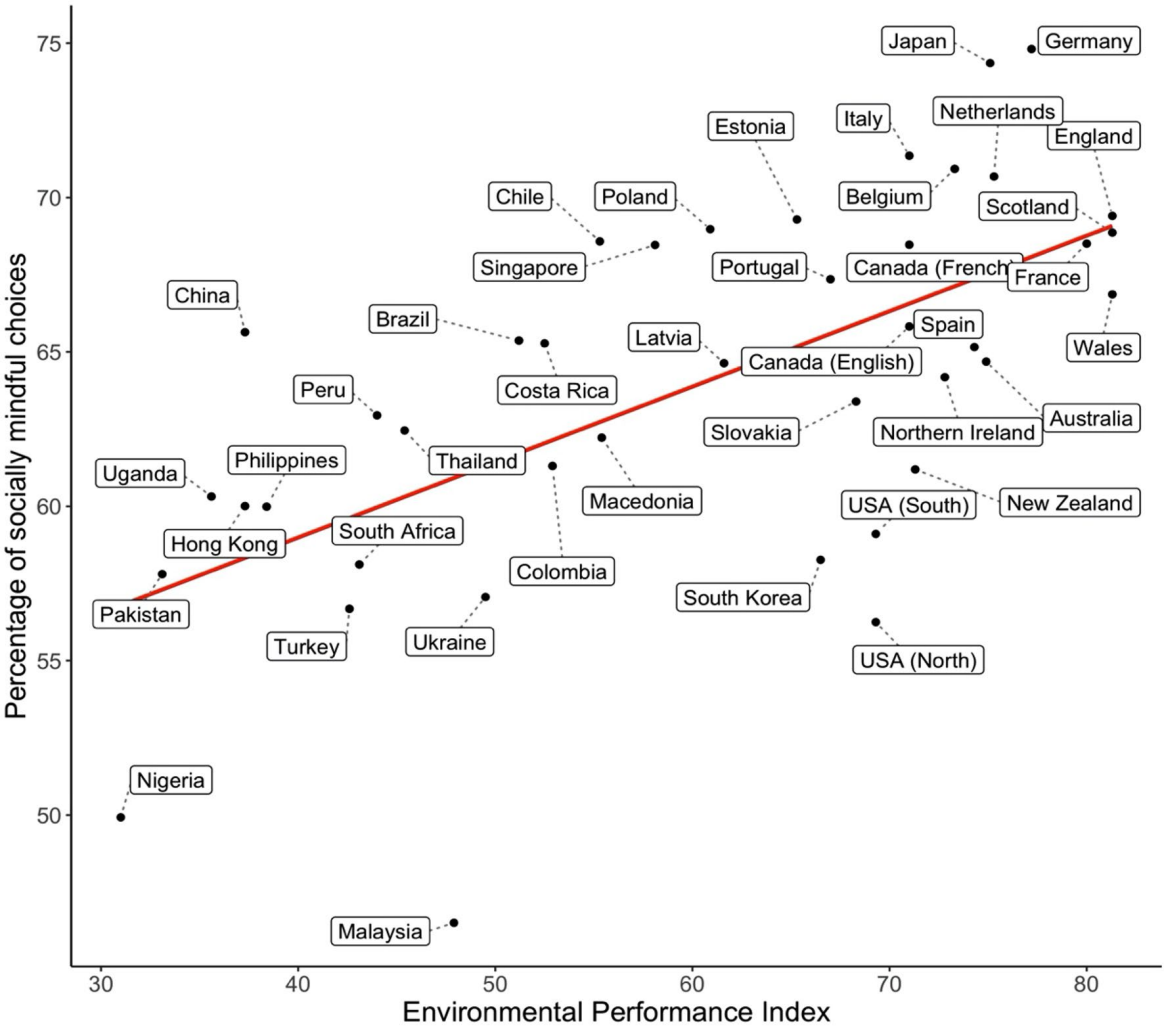
Relationship between the percentage of socially mindful choices and the Environmental Performance Index.

**Figure 3:**
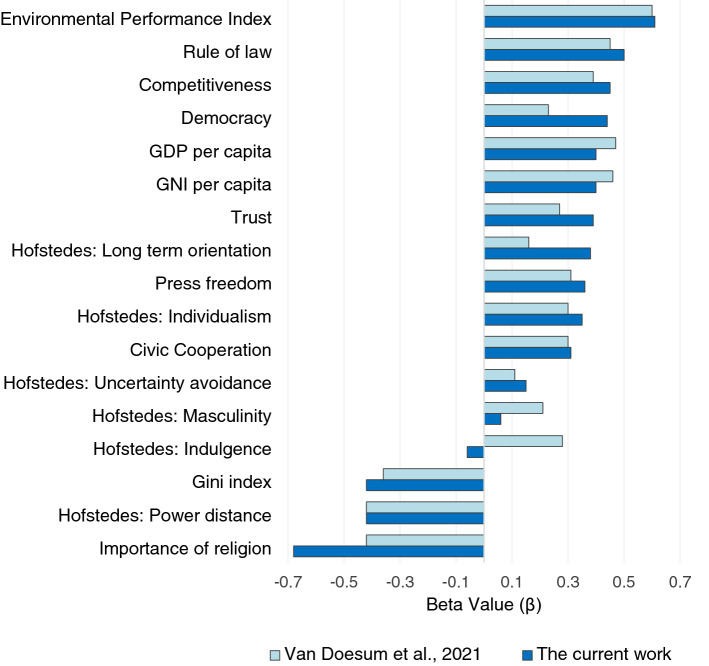
Comparison of Beta value (as an indicator of the effect size and direction of the effect) between the current work and Van Doesum and colleagues (2021). The Beta values can be interpreted in a similar way to Pearson coefficients. The findings for press freedom from the previous work^11^ was reverse scored for the sake of this comparison due to differences in coding.

We further analyzed the country-level bivariate relationships as outlined by Van Doesum and colleagues^11^ using seventeen Linear Mixed Models with sample location as the random intercept. The relationship between SoMi and (1) the key variables, (2) Hofstede’s dimensions and (3) economic indices, can be seen in Table 2. For each model, the variables were grand mean centered. More trust, lower rates of religion, a greater rule of law, more competitiveness, greater press freedom, lower power distance, more individualism, greater long-term orientation, a higher GDP, a higher GNI and a lower Gini coefficient were associated with more socially mindful choices. Critically, participants who made more socially mindful choices also came from countries with a higher EPI. See Fig. 2 for the relationship between EPI and SoMi per country. See Fig. 3 for a direct comparison of effect sizes between the current findings and the previous work^11^.

**Table 2:** Country-level bivariate relations with Somi across three domains.

Variables	β	*SE*	*p*
**Key variables**			
Trust	0.39	0.17	.026*
Religion	− 0.68	0.14	< .001***
Civic Cooperation	0.31	0.17	.081
Rule of law	0.50	0.14	< .001***
Democracy	0.44	0.14	.004**
Competitiveness	0.45	0.14	.004**
Press Freedom	0.36	0.15	.021*
EPI	0.61	0.13	< .001***
**Hofstede dimensions**			
Power Distance	− 0.42	0.15	.008**
Individualism	0.35	0.15	.030*
Masculinity	0.06	0.16	.706
Uncertainty avoidance	0.15	0.16	.376
Long term orientation	0.38	0.16	.019*
Indulgence	− 0.06	0.17	.720
**Economic indices**			
GNI per capita	0.40	0.15	.009**
GDP per capita	0.40	0.15	.009**
Gini	− 0.42	0.15	.007**

*p < .05, **p < .01, ***p < .001.

Overall, we replicated each of the country-level relationships from the study by Van Doesum and colleagues^11^ including a relationship between SoMi scores and religiosity, Rule of Law, Competitiveness Index, power distance, GNI and GDP. We additionally found evidence for several other relationships that were not significant in the prior work, including links between SoMi and country-level trust, democracy, individualism, long-term orientation, press freedom and the Gini index. Critically, we replicated the key relationship of interest: higher scores on the EPI were strongly associated with more socially mindful choices. Van Doesum and colleagues^11^ suggested that simple cooperative acts, such as the considerateness displayed in the SoMi measure, may foster the kinds of societies that engage in more complex forms of cooperation, such as protecting the environment. However, if this explanation is correct, SoMi should relate to other outcomes, such as those that encapsulate trust, positive community participation and more inclusive attitudes. To test this possibility, we explored the relationship between a simple, low-cost form of cooperation (SoMi) that is not embedded within a real-world context and more complex cooperative attitudes and outcomes that relate to current societal issues.

### Part 2: Exploratory examination of link between SoMi and complex cooperation

See Supplementary Materials 8 for full results of each model reported below and Supplementary Materials 9 for the within-country and between-country correlations for each of our variables. We examined several individual-level and country-level variables in separate Linear Mixed Models to assess the effect of SoMi on more complex forms of cooperative outcomes and attitudes, with sample location as the random intercept and four demographic control variables included in each model (age, gender, economic conservativism, and social conservativism). All individual-level variables were partitioned into a within-countries (country mean centered) and between-countries (grand mean centered for country averages) estimate, and country-level variables were grand mean centered. While there is much debate in the literature about when one should correct for multiple statistical tests^38^, we took a conservative approach and noted the Bonferroni corrected alpha cut-off value of *p* = .003. Full results for each model can be seen in Table 3. For the individual-level variables, we found multiple relationships within-countries; more socially mindful choices were related to greater moral expansiveness, higher moral concern for nature more specifically (i.e., the nature subscale of moral expansiveness), enhanced support for immigrants, greater adoption of universalism as a core value, reduced perceptions of anomie (and particularly anomie within the social fabric of society) and a greater adoption of benevolence as a core value. Between-countries, greater numbers of socially mindful choices were associated with more positive attitudes towards immigrants and reduced perceptions of anomie within the social fabric of society. However, we note that several of these relationships (i.e., universalism as a core value, anomie, and anomie within the social fabric) did not meet the Bonferroni corrected *p*-value cut-off and only reached significance when following the .05 alpha value. Based on advice from reviewers, we also conducted a Linear Mixed Model containing all nine country level predictors in the same model and detail of these results can be seen in Supplementary Materials 10.

**Table 3:** Relationship between social mindfulness and more complex forms of cooperative attitudes and outcomes.

Variables	Within-countries			Between-countries		
	β	*SE*	*p*	β	*SE*	*p*
**Individual level**						
Moral expansiveness	0.07	0.01	< .001***	− 0.02	0.16	.898
Moral expansiveness for nature	0.05	0.01	< .001***	− 0.15	0.16	.351
Negative attitudes towards immigrants	− 0.10	0.01	< .001***	− 0.58	0.13	< .001***
Schwartz universalism	0.03	0.01	.019*	− 0.07	0.16	.680
Anomie	− 0.03	0.01	.017*	− 0.21	0.15	.180
Anomie in the social fabric	− 0.03	0.01	.010*	− 0.33	0.15	.034*
Anomie in leadership	− 0.02	0.01	.112	− 0.11	0.16	.488
Schwartz benevolence	0.05	0.01	< .001***	− 0.04	0.16	.819
**Country level**						
Environmental care	–	–	–	0.41	0.15	.010*
Tolerance for minorities as neighbors	–	–	–	0.58	0.15	< .001***
Child quality: tolerance/respect	–	–	–	0.29	0.17	.101
Trust	–	–	–	0.33	0.16	.049*
Social capital index	–	–	–	0.58	0.13	< .001***
Collective action	–	–	–	0.47	0.16	.008*
Citizenship index	–	–	–	0.42	0.15	.008*
Voice and accountability of people	–	–	–	0.48	0.14	.002**
Child quality: unselfishness	–	–	–	0.14	0.18	.437

*p < .05, **p < .003^, ***p < .001.

Each variable was analyzed in a separate Linear Mixed Model with age, gender, economic conservatism, and social conservatism included as controls.

^Bonferroni corrected alpha cut-off.

For our country-level measures, higher SoMi scores were associated with greater environmental care, more positive attitudes towards having minority individuals as neighbors, a greater perception that most others can be trusted, a higher social capital index, more engagement in collective action, a higher citizenship index and greater voice and accountability of people. Several of these results remained robust when accounting for the Bonferroni adjustment, including a link between higher SoMi scores and positive attitudes towards having minority individuals as neighbors, a higher social capital index and greater voice and accountability of people. There was no clear relationship observed between SoMi and seeing ‘tolerance and respect’ or ‘unselfishness’ as an important child quality. With few exceptions, these results provide support that simple, low-cost forms of cooperation, such as that encapsulated by SoMi, is associated with a significant array of more complex cooperative outcomes and attitudes that relate to many of the current issues faced by society.

## Discussion

The capacity for cooperation is one of the key factors underlying well-functioning groups and societies^5,6^. Cooperation occurs in many forms, from considerateness towards the needs of another person, to more complex manifestations such as working together to protect the environment for current and future generations. Here we aimed to establish how socially mindful choices between a dyad – a context-free, low-cost form of cooperation – relate to more complex cooperative outcomes and attitudes. We replicated past work that proposed this link^11^ and examined how SoMi relates to factors that are linked to societal trust and positive community participation, and inclusive attitudes and moral obligation to protect others, including the environment. Overall, we found that SoMi was related to greater demonstrations of more complex cooperation measured on the individual level, such as more moral expansiveness and reduced prejudice towards immigrants. Critically, we found that the simple decision to leave another with choice was related to country objective indicators of complex cooperation including more social capital, greater tolerance for minorities and better environmental protection. Across these diverse measures, we found overwhelming evidence for our hypothesis with only a few exceptions.

Simple cooperative interactions, such as SoMi, may provide the bedrock for more complex forms of cooperation to grow^11,39,40^. If everyone in a society is considerate to the needs of others, this general attitude might help foster cooperative attitudes and outcomes that benefit all rather than just a few. For example, being considerate to the needs of strangers may result in more positive interactions with outgroup members, which in turn may foster more inclusive attitudes. Likewise, being more socially mindful of others may lead to more positive reciprocal interactions with strangers which in turn heightens trust that others will cooperate. The opposite direction is equally plausible; environments that are more broadly cooperative may nurture more positive dyadic interactions^10^. That is, societies that promote more inclusive attitudes towards others, and that foster trust and positive regard for others, may signal a cooperative social norm that results in individuals aligning their behaviors with that norm. In all likelihood, the relationship between simple, low-cost cooperation between a dyad and more complex cooperation is bidirectional and symbiotic. We also note that, when taking a conservative approach and adjusting for multiple tests, several relationships no longer significantly predicted SoMi, such as collective action, adopting a universalism value and perceiving anomie within society. However, a number of key results remained robust, and we showed a clear link between SoMi and support for minorities and immigrants, moral expansiveness, care for the environment and social capital. Future work may wish to further investigate whether SoMi is indeed linked to the variables that did not reach the conservative significance threshold, preferably in samples with greater numbers of countries to increase power.

This is the first work to showcase that the simple act of leaving another with choice is related to meaningful cooperative outcomes and many of these occur at the country level, such as environmental protection, better social capital, and more inclusive attitudes toward outgroup members. We first directly replicated past work^11^ in a novel sample with a greater variety of countries, demonstrating strong empirical ground for our investigation. The prediction that arose from this work was then directly tested across a variety of outcomes that broadly reflect complex forms of cooperation that link to many of the societal issues we currently face, such as the treatment of outgroup members, the climate crisis and community cohesion. While we have found substantial evidence that simple cooperative interactions (e.g., SoMi) relate to more complex forms of cooperation, we have also gained fascinating and novel insights into a range of specific relationships. For example, the link between socially mindful choices and positive support for outgroups such as immigrants, both on the individual level and country level, has been previously unknown (although see Manesi and colleagues^41^). The sheer diversity in these measures will no doubt result in a variety of future studies to further our understanding of cooperation.

Our work has raised several questions for future research. First, our study was correlational, making causality difficult to establish. Experimental work would confirm the directionality, and as we suspect, the bidirectionality of these relationships. For example, future work could manipulate how people react when they see networks of socially versus unsocially mindful individuals and see if this has spill over effects for eco-friendly behaviors. Additionally, our work came from a diverse range of countries and this approach combats the WEIRD bias in psychology^42^. However, each sample also came from a university and may not represent the demographics of each country. Future research could aim to achieve better representation. We also did not control for the participant’s preference for each object^43^, and past work has shown that people are less socially mindful when there is a greater opportunity cost to behaviour^44^. Future research may wish to explore how the cost of being socially mindful may differ depending on factors such as scarcity of resources or object preference.

We also found a relationship between individualism and greater SoMi and this result opposes suggestions from past work^45^. This may be because those in collectivist cultures tend to be more prosocial when there is opportunity for reciprocity and commitment whereas those in individualistic cultures prefer more spontaneous forms of prosociality^46^. For example, it is possible that prosociality in individualistic cultures is more strongly connected to prosociality toward strangers, whereas prosociality in collectivistic cultures is more strongly connected to members of ingroups affording greater reciprocity and commitment^47,48^. Given that SoMi focuses on strangers in its measurement, prosociality and individualism may be positively correlated. Future work may seek to explore whether those from collectivist cultures are instead more socially mindful when there are greater opportunities for reciprocity and the building of commitment. Finally, while we have assessed more complex forms of cooperation across many diverse measures, SoMi is only one example of a low cost, simple and context-free cooperative interaction. Future work could explore how other simple behaviors may relate to broader cooperative outcomes.

Cooperation is a part of everyday life, whether it is choosing to follow the road rules on our way to work, turning off the lights to help preserve the environment or leaving another with a choice of condiments at the breakfast buffet. These diverse examples of cooperation seem distinct at first blush, yet little research has explored whether these may be interrelated. Here we aimed to explore whether leaving another with choice (i.e., social mindfulness) relates to more complex forms of cooperation that relate to some of the more pressing issues of our time, such as (a) societal-level trust and positive community participation, and (b) inclusive attitudes and moral obligation to protect others, including the environment. Across 41 samples spanning 36 countries, we found that greater social mindfulness (SoMi) was related to a broad range of complex cooperative outcomes and attitudes, including greater environmental protection, better social capital, and more expansive moral concern.

## Supplementary Information

Supplementary Information.

## Data Availability

Our replication of the prior work was pre-registered on the Open Science Framework. Data will be available upon request from the corresponding author and all analysis code has been uploaded to the Open Science Framework (see: https://osf.io/452ur/?view_only=76022fb5424d45cda675a9859c62df51). All materials are available in Supplementary Materials.
