# Supplementary material for: Social mindfulness predicts concern for nature and immigrants across 36 nations
Source: Sci Rep. 2022 Dec 21;12:22102. doi: 10.1038/s41598-022-25538-y (PMC9772369; doi:10.1038/s41598-022-25538-y)
Supplement: Supplementary file 1 — Supplementary Information. [file 41598_2022_25538_MOESM1_ESM.pdf]

Supplementary Materials

Supplementary Materials 1: Information by Country 2
Supplementary Materials 2: SoMi Measure 4
Supplementary Materials 3: Individual-level variables for Part 1 8
Supplementary Materials 4: Country-level variables for Part 1 10
Supplementary Materials 5: Individual-level variables for Part 2 12
Supplementary Materials 6: Country-level variables for Part 2 16
Supplementary Materials 7: Full model results for Part 1 18
Supplementary Materials 8: Full model results for Part 2 29
Supplementary Materials 9: Within-Country and Between-Country Correlations for Part 2 40
Supplementary Materials 10: Results for Linear Mixed Model Containing all Country Level Variables for Part 2 43
References 45

Supplementary Materials 1: Information by Country

Table S1. Countries, survey language, data collection method and total responses.

Country	Survey Format	Sample Size	Language
Australia	Online	386	English
Belgium	Online	225	Dutch
Brazil	Online	138	Portuguese
Canada (English)	Online	183	English
Canada (French)	Online	135	French
Chile	Online	69	Spanish
China	Online	235	Chinese
Colombia	Online	151	Spanish
Costa Rica	Online	130	Spanish
England	Online	149	English
Estonia	Online	63	Estonian
France	Online	191	French
Germany	Online	143	German
Hong Kong	Online	211	English
Italy	Online	222	Italian
Japan	Online	182	Japanese
Latvia	Paper & Pencil	120	Latvian
Macedonia	Online	124	Macedonian
Malaysia	Online	175	Malay
Netherlands	Online	110	Dutch
New Zealand	Online	134	English
Nigeria	Paper & Pencil	233	English
Northern Ireland	Online	110	English
Pakistan	Online	147	English
Peru	Online	200	Spanish
Philippines	Online/Paper & Pencil	151	English
Poland	Paper & Pencil	116	Polish
Portugal	Online/Paper & Pencil	137	Portuguese
Scotland	Online	110	English
Singapore	Online	102	English
Slovakia	Paper & Pencil	115	Slovak
South Africa	Other(online)	423	English
South Korea	Other(online)	127	Korean
Spain	Online	156	Spanish

Thailand	Paper & Pencil	99	Thai
Turkey	Online	275	Turkish
Uganda	Online	101	English
Ukraine	Paper & Pencil	110	Ukrainian
USA (North)	Online	181	English
USA (South)	Online	211	English
Wales	Online	85	English

Supplementary Materials 2: SoMi Measure

[1]The task you are about to do involves two people; you and someone else. Imagine that the other person is someone you haven't met before, and will not knowingly meet again in the future (because you will not get to know or see each other in person).

Also imagine that you both get to choose one of the objects that are shown on the paper. There are only a few objects left. Once taken, these will not be replaced. In this task, you always get to choose first. You will have to make several choices.

So to summarize:

You and someone else can each choose one among the products shown on the paper.

It is important to remember that you always choose first!

➔ *Make your choice by circling the number under the product you would like to take.*

First, here's an example. Which of these objects would you take? You pick first, then the other!

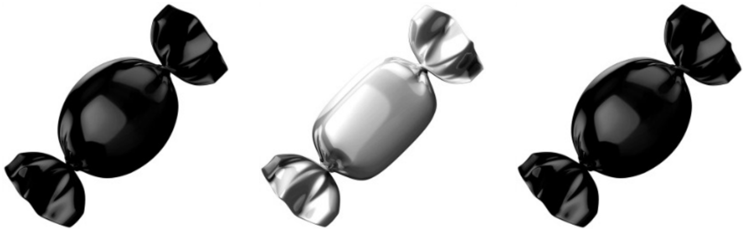

1 *2* *3*

Now turn the page to start the task.

Remember that there's two of you, and that you always choose first!
Which of these objects would you take? You pick first, then the other person!

1

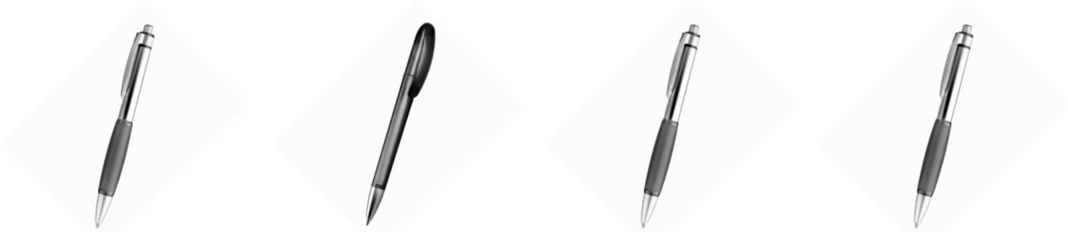

1 *2* *3* *4*

2

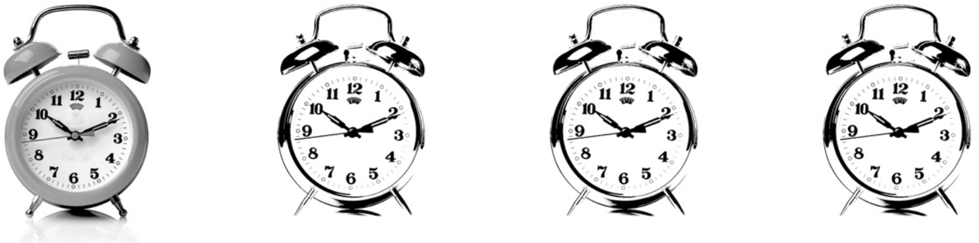

1 *2* *3* *4*

3

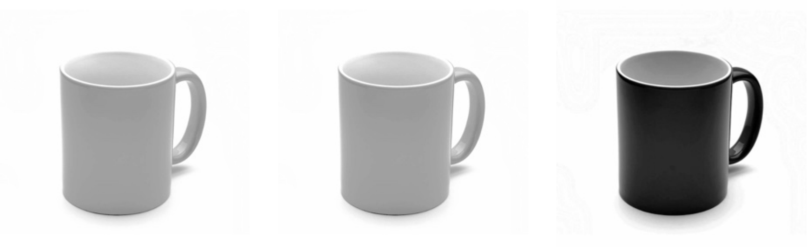

1 *2* *3*

4

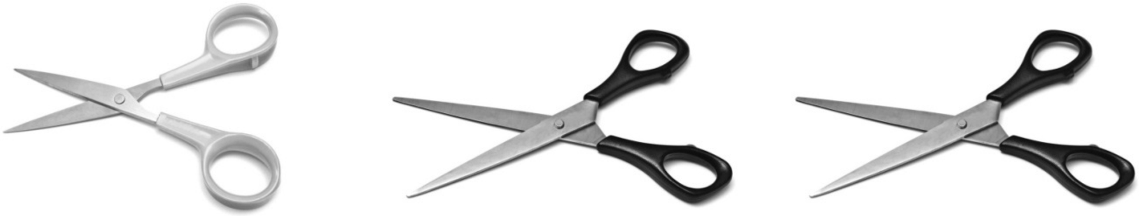

1 *2* *3*

5

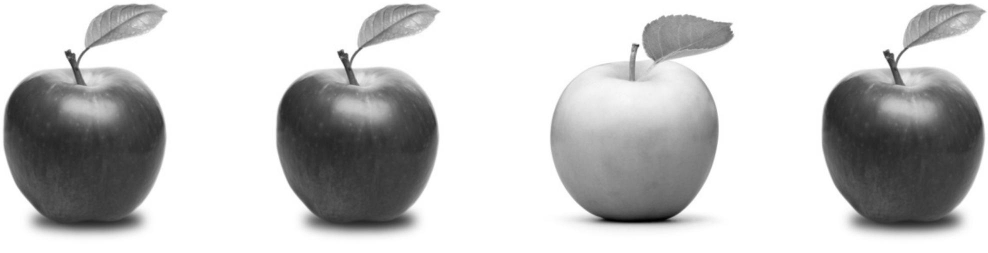

1 *2* *3* *4*

6

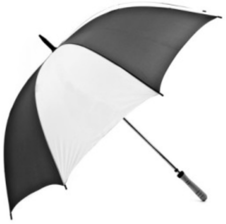

1

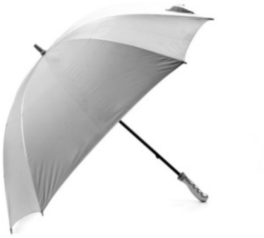

2

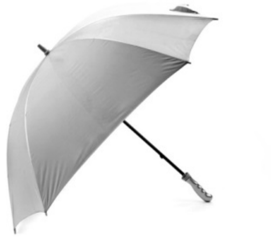

3

7

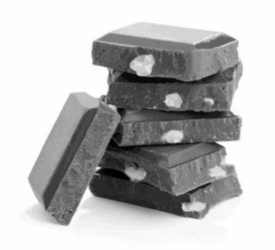

1

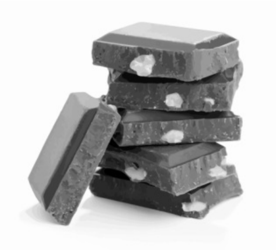

2

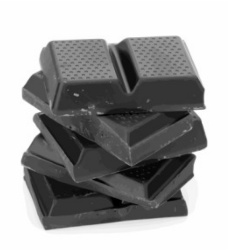

3

8

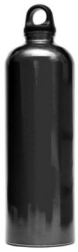

1

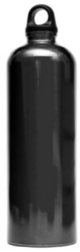

2

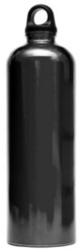

3

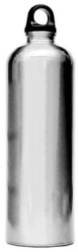

4

9

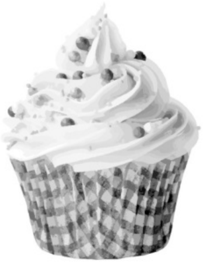

1

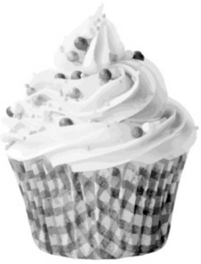

2

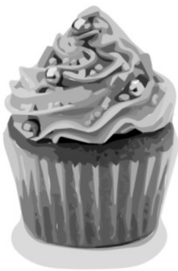

3

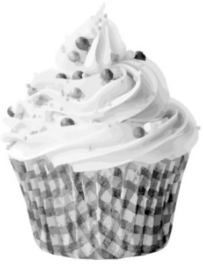

4

10

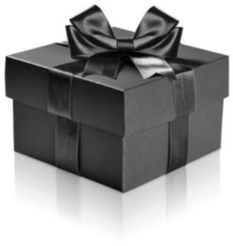

1

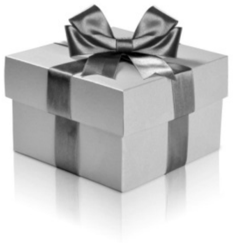

2

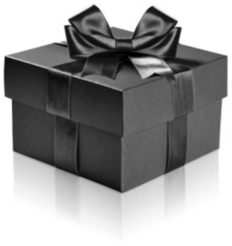

3

11

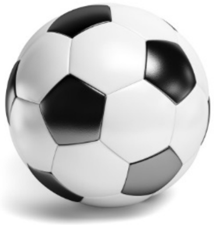

1

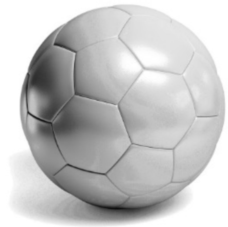

2

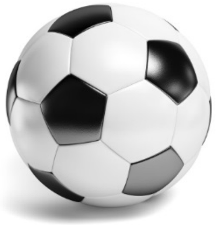

3

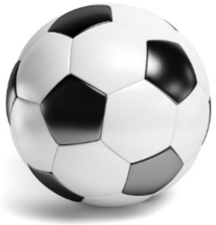

4

12

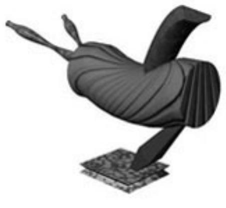

1

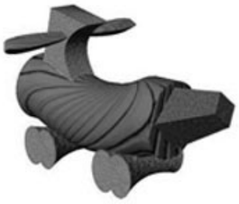

2

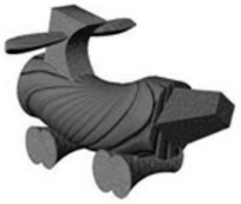

3

Supplementary Materials 3: Individual-level variables for Part 1

Age

- Age (in years): ______________

Gender

- Gender: ______________

Trust in others [2]

1. I dare to put my fate in the hands of most other people
2. I completely trust most other people
3. When push comes to shove, I do not trust most other people – reverse coded

Responses were recorded on a scale from 1 (*completely disagree*) to 7 (*completely agree*) and an average score for all items was calculated.

Others trust in you [2]

1. I think that most other people dare to put their fate in my hands
2. I think that most other people trust me
3. When push comes to shove, most other people do not trust me – reverse coded

Responses were recorded on a scale from 1 (*completely disagree*) to 7 (*completely agree*) and an average score for all items was calculated.

Economic conservatism

- Please indicate your political beliefs from left/liberal to right/conservative on issues of the economy (e.g., social welfare, government spending, tax cuts):

Responses were recorded on a scale from 1 (*left/liberal*) to 7 (*right/conservative*).

Social conservatism

- Please indicate your political beliefs from left/liberal to right/conservative on social issues (e.g., immigration, homosexual marriage, abortion):

Responses were recorded on a scale from 1 (*left/liberal*) to 7 (*right/conservative*)

MacArthur Scale of Subjective Socioeconomic Status [3]

- Think of this ladder as representing where people stand in <country>. At the top of the ladder are the people who have the most money, most education, and most respected jobs. At the bottom are the people who have the least money, least education, and least respected jobs or no job. The higher up you are on this ladder, the closer you are to the people at

the very top, and the lower you are, the closer you are to the people at the very bottom. Where would you place yourself on this ladder? Please select a number corresponding to the rung where you think you stand at this time in your life, relative to other people in <country>.

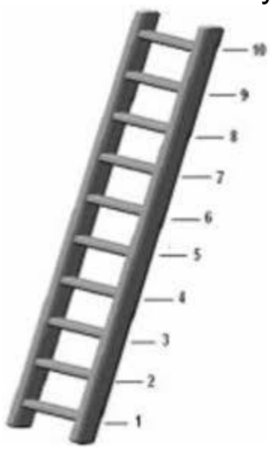

Responses were recorded on a scale from 1 (*least money, job prestige and education*) to 10 (*most money, job prestige and education*)

Supplementary Materials 4: Country-level variables for Part 1

Table S2. Country level variables and associated sources for Part 1

Variable	Description	Source
Trust	"Most people can be trusted" – recoded to 1 (*need to be very careful*) or 2 (*most people can be trusted)*	World Values Survey (Wave 6) [4] and European Values Survey (2017) [5]
Religiosity	"Important in life: Religion" – recoded to 1 (*not at all important*) to 4 (*very important*)	World Values Survey (Wave 6) [4] and European Values Survey (2017) [5]
Civic Cooperation	"How justifiable is it to: 1. Cheat on taxes 2. Not pay for a public bus fare 3. Claim government benefits you aren't entitled to" Average of responses from all three questions on a scale recoded to 1 (*always justifiable*) to 10 (*never justifiable*)	World Values Survey (Wave 6) [4] and European Values Survey (2017) [5]
Rule of Law	A measure for the confidence individuals have in the rules of society as well as the likelihood those individuals abide by those rules. Scores range from -2.5 (*weak rule of law*) to 2.5 (*strong rule of law*)	https://databank.worldbank.org/source/worldwide-governance-indicators/preview/on# [6]
Democracy	The state of democracy in the country, measuring pluralism, civil liberties and political culture. Measured from 0 (*least democratic*) to 10 (*most democratic*)	Economist Intelligence Unit https://www.eiu.com/n/campaigns/democracy-index-2020/ [7]
Global Competitiveness Index	A compilation of 12 facets of competitiveness. A scale from 0 (*less competitiveness*) to 100 (*more competitiveness*)	http://www3.weforum.org/docs/WEF_TheGlobalCompetitivenessReport2019.pdf [8]

World Press Freedom Index	Degree of freedom available to journalists in the country. Measured from 0 (*least freedom*) to 100 (*most freedom*)	https://rsf.org/en/index [9]
Environmenta l Performance Index (EPI)	Rank of performance on 24 environmental health indicators. Ranked from 0 (*worst performance*) to 100 (*best performance*)	http://epi.yale.edu [10]
Hofstede's Cultural Dimensions	Six cultural dimensions: Power Distance, Individualism, Masculinity, Uncertainty Avoidance, Long Term Orientation, and Indulgence	https://www.hofstede-insights.com/product/compare-countries/ [11]
GNI per capita	The income of a country per year divided by the number of people in that country in US$	https://data.worldbank.org/indicator/NY.GNP.PCAP.CD [12]
GDP per capita	The economic output of a country per year divided by the number of people in that country in US$	https://data.worldbank.org/indicator/NY.GDP.PCAP.CD [13]
Gini Index	A measure of economic inequality in a country. The index ranges from 0 (perfect equality) to 100 (perfect inequality)	https://www.cia.gov/the-world-factbook/field/gini-index-coefficient-distribution-of-family-income/country-comparison [14]

Supplementary Materials 5: Individual-level variables for Part 2

Moral Expansiveness Scale [15]

People sometimes talk about ***'circles of moral concern'***. These circles are simple ways to make sense of the *levels of moral consideration we have for different entities* (e.g., people, animals, and the environment).

Where we place these entities within our moral circles is important and has direct consequences for how we treat them. For example, you might have close family or friends that are central to your moral world, this means you would be willing to make personal sacrifices for them. However, if we do not include an entity within our moral circles, this means we do not believe they are deserving of moral care and consideration, and wouldn't want to make sacrifices for them.

On the following page you are given the opportunity to organise a range of entities and place them within your own moral circles that reflect your individual views and feelings.

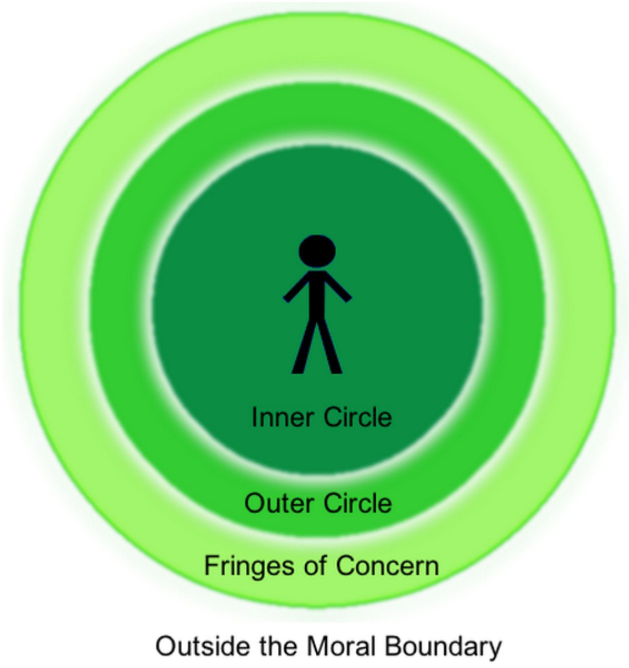

Please read the four moral boundary descriptions below before completing the moral circle task.

- ***Inner Circle of Moral Concern:*** These entities deserve the **highest level of moral concern and standing.** You have a moral obligation to ensure their welfare and feel a sense of personal responsibility for their treatment.

- ***Outer Circle of Moral Concern:*** These entities deserve **moderate moral concern and standing.** You are concerned about their moral treatment; however, your sense of obligation and personal responsibility is greatly reduced.

- ***Fringes of Moral Concern:*** These entities deserve **minimal moral concern and standing**, but you are not morally obligated or personally responsible for their moral treatment.

- ***Outside the Moral Boundary:*** These entities deserve **no moral concern or standing.** Feeling concern or personal responsibility for their moral treatment is extreme or nonsensical.

Having carefully read these descriptions, please consider the **level of moral concern you have for each of the entities below and select the appropriate moral circle placement.** Please note, there are no right or wrong answers – we just want to know your opinion.

- Rose bush
- Elected leader of your country (position, not specific individual)
- Close friend
- Mentally challenged individual
- Supporter of opposing political party
- Soldier of your country
- Fish
- Charity worker
- Terrorist
- Refugee
- Foreign citizen
- Chicken
- Partner/spouse
- Somebody with different religious beliefs
- Murderer
- Apple tree
- Co-worker
- Old-growth forest
- Bee
- Chimpanzee
- Homosexual
- Coral reef
- Redwood tree
- National park
- Somebody from your neighborhood
- Citizen of your country
- Cow

- Child molester
- Dolphin
- Family member

Responses are coded as 3 (*inner circle of moral concern*), 2 (*outer circle of moral concern*), 1 (*fringes of moral concern*) or 0 (*outside the moral boundary*). Responses to each entity are added together to create a total moral expansiveness score between 0 (*least morally expansive*) to 90 (*most morally expansive*).

<u>Attitudes towards immigrants</u> [16]

1. Immigrants take resources and employment opportunities away from <Country>
2. In schools where there are too many children of immigrants, the quality of education will suffer
3. Immigrants abuse the system of social benefits
4. <Country> norms and values are being threatened by the presence of immigrants
5. The cultural practices of immigrants threaten the <Country> way of life
6. Immigrants are a threat to the <Country> identity

Responses were recorded on a scale from 1 (*strongly disagree*) to 7 (*strongly agree*) and an average score for all items was calculated.

<u>Schwartz 'Universalism' Value</u> [17]

The next questionnaire asks about the things you <u>personally</u> value in life. Please answer in the space to the left of each item. First read the list of values below and choose the value that is most important to you and rate it **7**. Next, choose the value that is opposed to what you value and rate it **-1**. If there is no such value, choose the value least important to you and rate it **0** or **1**, according to its importance. **Then rate the rest of the values in the list:**

- Universalism, that is, equality, a world at peace, wisdom, social justice, broadmindedness, to enjoy the beauty of nature and the arts, to feel unity with nature and to protect the environment

Responses were recorded on a scale from -1 (*opposed to my principles*) to 7 (*very important*)

<u>Perceptions of anomie</u> [18,19]

- Social anomie
    1. People think that there are no clear moral standards to follow
    2. Everyone thinks of himself/herself and does not help others in need
    3. Most of people think that if something works, it doesn't really matter whether it is right or wrong
    4. People do not know who they can trust and rely on

5. Most of the people think that honesty doesn't work all the time; dishonesty is sometimes a better approach to get ahead
6. People are cooperative – reverse coded

- Government anomie
  1. The government works towards the welfare of people – reverse coded
  2. The government is legitimate – reverse coded
  3. The government uses its power legitimately – reverse coded
  4. Politicians don't care about the problems of average person
  5. The government laws and policies are effective – reverse coded
  6. Some laws are not fair

Responses were recorded on a scale from 1 (*strongly disagree*) to 7 (*strongly agree*) and an average score for all items was calculated to achieve a total anomie score. We calculated separate averages to achieve a social anomie and government anomie score.

Schwartz 'Benevolence' Value [17]
The next questionnaire asks about the things you personally value in life. Please answer in the space to the left of each item. First read the list of values below and choose the value that is most important to you and rate it **7**. Next, choose the value that is opposed to what you value and rate it **-1**. If there is no such value, choose the value least important to you and rate it **0** or **1**, according to its importance. **Then rate the rest of the values in the list:**

- Benevolence, that is, to be loyal, honest, helpful, responsible, and forgiving

Responses were recorded on a scale from -1 (*opposed to my principles*) to 7 (*very important*)

Supplementary Materials 6: Country-level variables for Part 2

Table S3. Country level variables and associated sources for Part 2

Variable	Description	Source
Environmental Care	A sub-measure of the Citizenship Index from the US News Best Country measures. This provides a score for how much care a country puts into the environment. Ranked from 0 (*worst performance*) to 100 (*best performance*)	https://www.usnews.com/news/best-countries/citizenship-rankings [20]
Minorities as neighbors	Participants asked what categories of people they would not like to have as neighbors from: people of a different race, heavy drinkers, immigrants/foreign workers, drug addicts, homosexuals. Responses are recorded for each category as 1 (mentioned) or 2 (not mentioned) and an average was obtained. Higher values indicate greater tolerance for minorities.	World Values Survey (Wave 6) [4] and European Values Survey (2017) [5]
Child quality: tolerance/respect	Participants are given a list of qualities children are encouraged to learn at home. They are asked to indicate five that they believe are important, including 'tolerance/respect' as an option. Recoded as 1 (*not mentioned*) or 2 (*mentioned*). Higher scores indicate greater value placed on this trait.	World Values Survey (Wave 6) [4] and European Values Survey (2017) [5]
Trust	Percentage of individuals who think most people can be trusted. Obtained from Our World in Data.	https://ourworldindata.org/trust [21]
Social Capital Index	A collated measure of five indices of social capital: health, equality, crime, freedom, and satisfaction. Higher values indicate more social capital. Obtained from the Global Sustainable Competitiveness Index.	https://solability.com/the-global-sustainable-competitiveness-index/the-index/social-capital [22]

Citizenship Index	From the US News Best Country measures. The Citizenship Index provides a measure of positive global citizenship by collating the following measures: well-distributed political power, cares about the environment, gender equality, cares about human rights, progressive, respects property rights, religious freedom, trustworthy. Ranked from 0 (*worst performance*) to 100 (*best performance*)	https://www.usnews.com/news/best-countries/citizenship-rankings [20]
Voice & Accountability of people	A worldwide governance indicator subscale from the World Bank. The measure assesses citizen participation in government and freedom of expression. Scores approximately range between -2 (*worst performance*) to 2 (*best performance*)	http://info.worldbank.org/governance/wgi/ [23]
Collective action	Have participants engaged in these behaviors:<br>1. Signed a petition<br>2. Attended a peaceful protest<br>Average of responses from both questions were recoded on a scale from 1 (*would never do*), 2 (*might do*) 3 (*have done*). Higher values indicate more collective action.	World Values Survey (Wave 6) [4] and European Values Survey (2017) [5]
Child quality: unselfishness	Participants are given a list of qualities children are encouraged to learn at home. They are asked to indicate five that they believe are important, including ‘unselfishness’ as an option. Recoded as 1 (*not mentioned*) or 2 (*mentioned*). Higher scores indicate greater value placed on this trait.	World Values Survey (Wave 6) [4] and European Values Survey (2017) [5]

Supplementary Materials 7: Full model results for Part 1

Table S4. Relationship between social mindfulness and age.

	Social Mindfulness		
Predictors	*Estimates*	*CI*	*p*
(Intercept)	-0.00	-0.32, 0.32	.992
Age (within countries)	0.01	-0.01, 0.04	.295
Age (between countries)	0.12	-0.20, 0.44	.460
**Random Effects**			
Residual	1.00		
Country (intercept)	1.00		
ICC	.50		
N (Country)	41		
Observations	6153		
Marginal R^2 / Conditional R^2	.006 / .505		

Table S5. Relationship between social mindfulness and subjective social status.

	Social Mindfulness		
Predictors	*Estimates*	*CI*	*p*
(Intercept)	0.00	-0.31, 0.32	.989
Subjective social status (within countries)	0.00	-0.02, 0.03	.773
Subjective social status (between countries)	0.17	-0.15, 0.49	.283
**Random Effects**			
Residual	1.00		
Country (intercept)	0.99		
ICC	.50		
N (Country)	41		
Observations	6125		
Marginal R^2 / Conditional R^2	.015 / .507		

Table S6. Relationship between social mindfulness and gender.

	Social Mindfulness		
Predictors	*Estimates*	*CI*	*p*
(Intercept)	0.03	-0.24, 0.30	.817
Gender (within countries)	-0.01	-0.03, 0.02	.543
Gender (between countries)	0.61	0.33, 0.89	<.001
**Random Effects**			
Residual	1.00		
Country (intercept)	0.68		
ICC	.41		
N (Country)	40		
Observations	6055		
Marginal R^2 / Conditional R^2	.203 / .527		

Table S7. Relationship between social mindfulness and economic conservativism.

	Social Mindfulness		
Predictors	*Estimates*	*CI*	*p*
(Intercept)	-0.00	-0.28, 0.28	.988
Economic conservativism (within countries)	-0.05	-0.07, -0.02	<.001
Economic conservativism (between countries)	-0.50	-0.78, -0.21	.001
**Random Effects**			
Residual	1.00		
Country (intercept)	0.77		
ICC	.44		
N (Country)	41		
Observations	6143		
Marginal R^2 / Conditional R^2	.120 / .503		

Table S8. Relationship between social mindfulness and social conservativism.

	Social Mindfulness		
Predictors	*Estimates*	*CI*	*p*
(Intercept)	-0.00	-0.25, 0.25	0.987
Social conservativism (within countries)	-0.03	-0.06, -0.01	.010
Social conservativism (between countries)	-0.63	-0.88, -0.38	<.001
**Random Effects**			
Residual	1.00		
Country (intercept)	0.61		
ICC	.38		
N (Country)	41		
Observations	6142		
Marginal R^2 / Conditional R^2	.182 / .493		

Table S9. Relationship between social mindfulness and trust in others.

	Social Mindfulness		
Predictors	*Estimates*	*CI*	*p*
(Intercept)	0.00	-0.31, 0.32	.993
Trust in others (within countries)	0.05	0.02, 0.07	<.001
Trust in others (between countries)	0.20	-0.12, 0.52	.210
**Random Effects**			
Residual	0.99		
Country (intercept)	0.98		
ICC	.50		
N (Country)	41		
Observations	6176		
Marginal R^2 / Conditional R^2	.022 / .507		

Table S10. Relationship between social mindfulness and others trust in self.

	Social Mindfulness		
Predictors	*Estimates*	*CI*	*p*

(Intercept)	0.00	-0.31, 0.32	.991
Others trust in self (within countries)	0.03	0.00, 0.05	.037
Others trust in self (between countries)	0.19	-0.13, 0.50	.244
**Random Effects**			
Residual	1.00		
Country (intercept)	0.98		
ICC	.50		
N (Country)	41		
Observations	6173		
Marginal R^2 / Conditional R^2	.017 / .505		

Table S11. Relationship between social mindfulness and trust (WVS).

	Social Mindfulness		
Predictors	*Estimates*	*CI*	*p*
(Intercept)	-0.06	-0.40, 0.28	.733
Trust (WVS)	0.39	0.05, 0.74	.026
**Random Effects**			
Residual	1.00		
Country (intercept)	0.97		
ICC	.49		
N (Country)	35		
Observations	5367		
Marginal R^2 / Conditional R^2	.076 / .530		

Table S12. Relationship between social mindfulness and importance of religion.

	Social Mindfulness		
Predictors	*Estimates*	*CI*	*p*
(Intercept)	-0.06	-0.34, 0.22	.677
Religiosity	-0.68	-0.97, -0.40	<.001

**Random Effects**	
Residual	1.00
Country (intercept)	0.65
ICC	.39
N (Country)	35
Observations	5367
Marginal R^2 / Conditional R^2	.230 / .533

Table S13. Relationship between social mindfulness and civic cooperation.

	**Social Mindfulness**		
Predictors	*Estimates*	*CI*	*p*
(Intercept)	-0.06	-0.41, 0.29	.741
Civic Cooperation	0.31	-0.04, 0.67	.080
**Random Effects**			
Residual	1.00		
Country (intercept)	1.03		
ICC	.51		
N (Country)	35		
Observations	5367		
Marginal R^2 / Conditional R^2	.065 / .539		

Table S14. Relationship between social mindfulness and rule of law.

	**Social Mindfulness**		
Predictors	*Estimates*	*CI*	*p*
(Intercept)	-0.00	-0.28, 0.28	.994
Rule of Law	0.50	0.22, 0.78	.001
**Random Effects**			
Residual	1.00		
Country (intercept)	0.76		
ICC	.43		

N (Country)	41
Observations	6190
Marginal R^2 / Conditional R^2	.127 / .505

Table S15. Relationship between social mindfulness and democracy.

	Social Mindfulness		
Predictors	*Estimates*	*CI*	*p*
(Intercept)	-0.00	-0.29, 0.29	.995
Democracy	0.44	0.14, 0.73	.004
**Random Effects**			
Residual	1.00		
Country (intercept)	0.82		
ICC	.45		
N (Country)	41		
Observations	6190		
Marginal R^2 / Conditional R^2	.109 / .512		

Table S16. Relationship between social mindfulness and competitiveness.

	Social Mindfulness		
Predictors	*Estimates*	*CI*	*p*
(Intercept)	-0.00	-0.29, 0.29	.993
Competitiveness Index	0.45	0.15, 0.74	.004
**Random Effects**			
Residual	1.00		
Country (intercept)	0.82		
ICC	.45		
N (Country)	41		
Observations	6190		
Marginal R^2 / Conditional R^2	.095 / .502		

Table S17. Relationship between social mindfulness and press freedom.

	Social Mindfulness		
Predictors	*Estimates*	*CI*	*p*
(Intercept)	-0.00	-0.30, 0.30	.996
Press Freedom	0.36	0.06, 0.66	.021
**Random Effects**			
Residual	1.00		
Country (intercept)	0.89		
ICC	.47		
N (Country)	41		
Observations	6190		
Marginal R^2 / Conditional R^2	.073 / .509		

Table S18. Relationship between social mindfulness and EPI.

	Social Mindfulness		
Predictors	*Estimates*	*CI*	*p*
(Intercept)	-0.00	-0.25, 0.25	.993
Environmental Performance Index	0.61	0.36, 0.87	<.001
**Random Effects**			
Residual	1.00		
Country (intercept)	0.64		
ICC	.39		
N (Country)	41		
Observations	6190		
Marginal R^2 / Conditional R^2	.195 / .508		

Table S19. Relationship between social mindfulness and Hofstede's power distance.

	Social Mindfulness		
Predictors	*Estimates*	*CI*	*p*

(Intercept)	0.01	-0.28, 0.31	.925
Hofstede's power distance	-0.42	-0.72, -0.12	.008
**Random Effects**			
Residual	1.00		
Country (intercept)	0.86		
ICC	.46		
N (Country)	40		
Observations	6127		
Marginal R^2 / Conditional R^2	.079 / .504		

Table S20. Relationship between social mindfulness and Hofstede's individualism.

	**Social Mindfulness**		
Predictors	*Estimates*	*CI*	*p*
(Intercept)	0.01	-0.29, 0.32	.928
Hofstede's individualism	0.35	0.04, 0.66	.030
**Random Effects**			
Residual	1.00		
Country (intercept)	0.91		
ICC	.48		
N (Country)	40		
Observations	6127		
Marginal R^2 / Conditional R^2	.057 / .507		

Table S21. Relationship between social mindfulness and Hofstede's masculinity.

	**Social Mindfulness**		
Predictors	*Estimates*	*CI*	*p*
(Intercept)	0.01	-0.31, 0.34	.931
Hofstede's masculinity	0.06	-0.27, 0.39	.706
**Random Effects**			

Residual	1.00
Country (intercept)	1.03
ICC	.51
N (Country)	40
Observations	6127
Marginal R^2 / Conditional R^2	.002 / .509

Table S22. Relationship between social mindfulness and Hofstede's uncertainty avoidance.

	Social Mindfulness		
Predictors	*Estimates*	*CI*	*p*
(Intercept)	0.01	-0.31, 0.34	.930
Hofstede's uncertainty avoidance	0.15	-0.18, 0.47	.376
**Random Effects**			
Residual	1.00		
Country (intercept)	1.02		
ICC	.50		
N (Country)	40		
Observations	6127		
Marginal R^2 / Conditional R^2	.009 / .509		

Table S23. Relationship between social mindfulness and Hofstede's long-term orientation.

	Social Mindfulness		
Predictors	*Estimates*	*CI*	*p*
(Intercept)	0.01	-0.30, 0.32	.954
Hofstede's long-term orientation	0.38	0.07, 0.70	.019
**Random Effects**			
Residual	1.00		
Country (intercept)	0.91		
ICC	.48		

N (Country)	39
Observations	6001
Marginal R^2 / Conditional R^2	.072 / .515

Table S24. Relationship between social mindfulness and Hofstede's indulgence.

	Social Mindfulness		
Predictors	*Estimates*	*CI*	*p*
(Intercept)	0.01	-0.33, 0.34	.958
Hofstede's indulgence	-0.06	-0.40, 0.28	.720
**Random Effects**			
Residual	1.00		
Country (intercept)	1.06		
ICC	.51		
N (Country)	39		
Observations	6001		
Marginal R^2 / Conditional R^2	.002 / .515		

Table S25. Relationship between social mindfulness and GNI per capita.

	Social Mindfulness		
Predictors	*Estimates*	*CI*	*p*
(Intercept)	-0.00	-0.29, 0.29	.993
GNI Per Capita	0.40	0.11, 0.70	.009
**Random Effects**			
Residual	1.00		
Country (intercept)	0.85		
ICC	.46		
N (Country)	41		
Observations	6190		
Marginal R^2 / Conditional R^2	.086 / .507		

Table S26. Relationship between social mindfulness and GDP per capita.

	**Social Mindfulness**		
Predictors	*Estimates*	*CI*	*p*
(Intercept)	-0.00	-0.29, 0.29	.993
GDP Per Capita	0.40	0.10, 0.70	.009
**Random Effects**			
Residual	1.00		
Country (intercept)	0.85		
ICC	.46		
N (Country)	41		
Observations	6190		
Marginal R^2 / Conditional R^2	.085 / .506		

Table S27. Relationship between social mindfulness and Gini index.

	**Social Mindfulness**		
Predictors	*Estimates*	*CI*	*p*
(Intercept)	-0.00	-0.29, 0.29	.996
Gini Index	-0.42	-0.71, -0.12	.007
**Random Effects**			
Residual	1.00		
Country (intercept)	0.84		
ICC	.46		
N (Country)	41		
Observations	6190		
Marginal R^2 / Conditional R^2	.104 / .514		

Supplementary Materials 8: Full model results for Part 2

Table S28. Relationship between social mindfulness and moral expansiveness.

	Social Mindfulness		
Predictors	*Estimates*	*CI*	*p*
(Intercept)	0.01	-0.31, 0.33	.940
Moral expansiveness (between countries)	-0.02	-0.34, 0.30	.898
Moral expansiveness (within countries)	0.07	0.05, 0.10	<.001
Age	0.01	-0.02, 0.04	.383
Gender (female)	-0.02	-0.08, 0.04	.447
Social conservativism	-0.01	-0.04, 0.02	.459
Economic conservativism	-0.03	-0.07, -0.00	.032
**Random Effects**			
Residual	0.99		
Country (intercept)	1.01		
ICC	.50		
N (Country)	41		
Observations	6000		
Marginal R^2 / Conditional R^2	.004 / .505		

Table S29. Relationship between social mindfulness and moral expansiveness for nature.

	Social Mindfulness		
Predictors	*Estimates*	*CI*	*p*
(Intercept)	0.01	-0.31, 0.33	.950
Moral expansiveness for nature (between countries)	-0.15	-0.47, 0.17	.351
Moral expansiveness for nature (within countries)	0.05	0.02, 0.07	<.001
Age	0.02	-0.02, 0.05	.324

Gender (female)	-0.02	-0.08, 0.04	.513
Social conservativism	-0.01	-0.04, 0.02	.452
Economic conservativism	-0.04	-0.07, -0.00	.023
**Random Effects**			
Residual	1.00		
Country (intercept)	0.98		
ICC	.49		
N (Country)	41		
Observations	6052		
Marginal R^2 / Conditional R^2	.012 / .501		

Table S30. Relationship between social mindfulness and negative attitudes towards immigrants.

	Social Mindfulness		
Predictors	*Estimates*	*CI*	*p*
(Intercept)	0.01	-0.25, 0.28	.922
Attitudes towards immigrants (between countries)	-0.58	-0.85, -0.32	<.001
Attitudes towards immigrants (within countries)	-0.10	-0.12, -0.07	<.001
Age	0.02	-0.01, 0.05	.219
Gender (female)	-0.02	-0.08, 0.03	.428
Social conservativism	0.01	-0.02, 0.05	.415
Economic conservativism	-0.02	-0.05, 0.01	.136
**Random Effects**			
Residual	0.99		
Country (intercept)	0.67		
ICC	.40		
N (Country)	41		
Observations	6080		

Marginal R^2 / Conditional R^2	.182 / .512

Table S31. Relationship between social mindfulness and Schwarz universalism value.

	Social Mindfulness		
Predictors	*Estimates*	*CI*	*p*
(Intercept)	0.02	-0.30, 0.33	.922
Universalism value (between countries)	-0.07	-0.39, 0.25	.679
Universalism value (within countries)	0.03	0.00, 0.06	.019
Age	0.02	-0.01, 0.05	.262
Gender (female)	-0.02	-0.08, 0.03	.406
Social conservativism	-0.01	-0.04, 0.02	.524
Economic conservativism	-0.03	-0.07, -0.00	.030
**Random Effects**			
Residual	1.00		
Country (intercept)	1.00		
ICC	.50		
N (Country)	41		
Observations	6066		
Marginal R^2 / Conditional R^2	.003 / .502		

Table S32. Relationship between social mindfulness and anomie.

	Social Mindfulness		
Predictors	*Estimates*	*CI*	*p*
(Intercept)	0.01	-0.30, 0.32	.958
Anomie (between countries)	-0.21	-0.52, 0.10	.180
Anomie (within countries)	-0.03	-0.06, -0.01	.017
Age	0.02	-0.01, 0.05	.301
Gender (female)	-0.02	-0.08, 0.04	.583

Social conservativism	-0.01	-0.04, 0.02	.419
Economic conservativism	-0.04	-0.07, -0.01	.013
**Random Effects**			
Residual	1.00		
Country (intercept)	0.95		
ICC	.49		
N (Country)	41		
Observations	6055		
Marginal R^2 / Conditional R^2	.023 / .500		

Table S33. Relationship between social mindfulness and anomie in the social fabric.

	Social Mindfulness		
Predictors	*Estimates*	*CI*	*p*
(Intercept)	0.01	-0.29, 0.31	.957
Anomie within the social fabric (between countries)	-0.33	-0.63, -0.03	.034
Anomie within the social fabric (within countries)	-0.03	-0.06, -0.01	.010
Age	0.02	-0.01, 0.05	.292
Gender (female)	-0.02	-0.08, 0.04	.586
Social conservativism	-0.01	-0.04, 0.02	.494
Economic conservativism	-0.04	-0.07, -0.01	.015
**Random Effects**			
Residual	1.00		
Country (intercept)	0.89		
ICC	.47		
N (Country)	41		
Observations	6071		
Marginal R^2 / Conditional R^2	.057 / .501		

Table S34. Relationship between social mindfulness and anomie in leadership.

	Social Mindfulness		
Predictors	*Estimates*	*CI*	*p*
(Intercept)	0.01	-0.31, 0.33	.961
Anomie within leadership (between countries)	-0.11	-0.43, 0.21	.488
Anomie within leadership (within countries)	-0.02	-0.05, 0.00	.112
Age	0.02	-0.01, 0.05	.273
Gender (female)	-0.02	-0.08, 0.04	.565
Social conservativism	-0.01	-0.05, 0.02	.385
Economic conservativism	-0.04	-0.07, -0.01	.012
**Random Effects**			
Residual	1.00		
Country (intercept)	0.98		
ICC	.50		
N (Country)	41		
Observations	6069		
Marginal R^2 / Conditional R^2	.007 / .500		

Table S35. Relationship between social mindfulness and Schwarz benevolence value.

	Social Mindfulness		
Predictors	*Estimates*	*CI*	*p*
(Intercept)	0.01	-0.31, 0.33	.928
Benevolence value (between countries)	-0.04	-0.36, 0.28	.819
Benevolence value (within countries)	0.05	0.03, 0.08	<.001
Age	0.02	-0.01, 0.05	.308
Gender (female)	-0.03	-0.08, 0.03	.404

Social conservativism	-0.02	-0.05, 0.02	.333
Economic conservativism	-0.04	-0.07, -0.00	.025
**Random Effects**			
Residual	0.99		
Country (intercept)	1.00		
ICC	.50		
N (Country)	41		
Observations	6056		
Marginal R^2 / Conditional R^2	.003 / .503		

Table S36. Relationship between social mindfulness and environmental care.

	Social Mindfulness		
Predictors	*Estimates*	*CI*	*p*
(Intercept)	0.11	-0.19, 0.41	.463
Environmental care	0.41	0.10, 0.71	.010
Age	0.01	-0.02, 0.04	.455
Gender (female)	-0.02	-0.08, 0.05	.570
Social conservativism	-0.03	-0.07, 0.00	.060
Economic conservativism	-0.04	-0.07, -0.00	.036
**Random Effects**			
Residual	1.00		
Country (intercept)	0.74		
ICC	.43		
N (Country)	35		
Observations	5154		
Marginal R^2 / Conditional R^2	.098 / .482		

Table S37. Relationship between social mindfulness and tolerance for minorities as neighbors.

	Social Mindfulness		
Predictors	*Estimates*	*CI*	*p*
(Intercept)	-0.05	-0.35, 0.26	.755
Tolerance for minorities	0.58	0.27, 0.88	.001
Age	0.03	-0.01, 0.06	.108
Gender (female)	-0.02	-0.09, 0.04	.481
Social conservativism	-0.02	-0.06, 0.01	.178
Economic conservativism	-0.03	-0.06, 0.00	.066
**Random Effects**			
Residual	1.00		
Country (intercept)	0.76		
ICC	.43		
N (Country)	35		
Observations	5278		
Marginal R^2 / Conditional R^2	.151 / .517		

Table S38. Relationship between social mindfulness and valuing tolerance/respect as a child quality.

	Social Mindfulness		
Predictors	*Estimates*	*CI*	*p*
(Intercept)	-0.05	-0.40, 0.30	.790
Child quality: tolerance/respect	0.29	-0.06, 0.65	.101
Age	0.03	-0.01, 0.06	.101
Gender (female)	-0.02	-0.09, 0.04	.479
Social conservativism	-0.02	-0.06, 0.01	.181
Economic conservativism	-0.03	-0.06, 0.00	.065
**Random Effects**			
Residual	1.00		
Country (intercept)	1.01		

ICC	.50
N (Country)	35
Observations	5278
Marginal R^2 / Conditional R^2	.042 / .525

Table S39. Relationship between social mindfulness and trust.

	**Social Mindfulness**		
Predictors	*Estimates*	*CI*	*p*
(Intercept)	-0.04	-0.36, 0.29	.828
Trust	0.33	0.00, 0.66	.049
Age	0.02	-0.01, 0.05	.261
Gender (female)	-0.01	-0.07, 0.05	.751
Social conservativism	-0.02	-0.05, 0.02	.320
Economic conservativism	-0.04	-0.07, -0.01	.011
**Random Effects**			
Residual	1.00		
Country (intercept)	0.94		
ICC	.48		
N (Country)	37		
Observations	5576		
Marginal R^2 / Conditional R^2	.058 / .515		

Table S40. Relationship between social mindfulness and social capital index.

	**Social Mindfulness**		
Predictors	*Estimates*	*CI*	*p*
(Intercept)	0.01	-0.25, 0.27	.948
Social Capital Index	0.58	0.31, 0.84	<.001
Age	0.02	-0.01, 0.05	.258
Gender (female)	-0.02	-0.08, 0.04	.534

Social conservativism	-0.01	-0.04, 0.02	.418
Economic conservativism	-0.04	-0.07, -0.01	.016
**Random Effects**			
Residual	1.00		
Country (intercept)	0.66		
ICC	.40		
N (Country)	41		
Observations	6089		
Marginal R^2 / Conditional R^2	.181 / .506		

Table S41. Relationship between social mindfulness and collective action.

	Social Mindfulness		
Predictors	*Estimates*	*CI*	*p*
(Intercept)	-0.07	-0.40, 0.27	.684
Collective action	0.47	0.13, 0.80	.008
Age	0.03	-0.00, 0.06	.081
Gender (female)	-0.03	-0.09, 0.03	.375
Social conservativism	-0.02	-0.06, 0.01	.153
Economic conservativism	-0.03	-0.06, 0.00	.093
**Random Effects**			
Residual	1.00		
Country (intercept)	0.89		
ICC	.47		
N (Country)	34		
Observations	5176		
Marginal R^2 / Conditional R^2	.114 / .531		

Table S42. Relationship between social mindfulness and citizenship index.

Social Mindfulness

Predictors	*Estimates*	*CI*	*p*
(Intercept)	0.11	-0.19, 0.41	.460
Citizenship index	0.42	0.12, 0.72	.008
Age	0.01	-0.02, 0.04	.461
Gender (female)	-0.02	-0.08, 0.05	.567
Social conservativism	-0.03	-0.07, 0.00	.061
Economic conservativism	-0.04	-0.07, -0.00	.036
**Random Effects**			
Residual	1.00		
Country (intercept)	0.73		
ICC	.42		
N (Country)	35		
Observations	5154		
Marginal R^2 / Conditional R^2	.097 / .479		

Table S43. Relationship between social mindfulness and voice and accountability of the people.

	**Social Mindfulness**		
Predictors	*Estimates*	*CI*	*p*
(Intercept)	-0.01	-0.30, 0.28	.947
Voice and accountability of people	0.48	0.20, 0.77	.002
Age	0.02	-0.02, 0.05	.319
Gender (female)	-0.02	-0.08, 0.04	.611
Social conservativism	-0.02	-0.05, 0.02	.358
Economic conservativism	-0.04	-0.07, -0.01	.012
**Random Effects**			
Residual	1.00		
Country (intercept)	0.76		
ICC	.43		

N (Country)	39
Observations	5811
Marginal R^2 / Conditional R^2	.130 / .507

Table S44. Relationship between social mindfulness and valuing unselfishness as a child quality.

	**Social Mindfulness**		
Predictors	*Estimates*	*CI*	*p*
(Intercept)	-0.05	-0.41, 0.31	.795
Child quality: Unselfishness	0.14	-0.22, 0.51	.437
Age	0.03	-0.01, 0.06	.100
Gender (female)	-0.02	-0.09, 0.04	.483
Social conservativism	-0.02	-0.06, 0.01	.178
Economic conservativism	-0.03	-0.06, 0.00	.065
**Random Effects**			
Residual	1.00		
Country (intercept)	1.08		
ICC	.52		
N (Country)	35		
Observations	5278		
Marginal R^2 / Conditional R^2	.010 / .525		

Supplementary Materials 9: Within-Country and Between-Country Correlations for Part 2

Table S45. Within-country correlations

Variable	*1*	*2*	*3*	*4*	*5*	*6*	*7*	*8*	*9*
1. Social Mindfulness	1.00								
2. MES	0.07***	1.00							
3. MES environment	0.05***	0.81***	1.00						
4. Attitude toward immigrants	-0.09***	-0.08***	-0.02	1.00					
5. Universalism value	0.04**	0.17***	0.13***	-0.17***	1.00				
6. Anomie	-0.03*	-0.02	0.04**	0.03**	0.06***	1.00			
7. Anomie in social fabric	-0.04**	-0.03*	0.01	0.11***	0.04**	0.79***	1.00		
8. Anomie in leadership	-0.02	-0.00	0.05***	-0.05***	0.06***	0.82***	0.31***	1.00	
9. Benevolence value	0.05***	0.06***	0.01	-0.08***	0.42***	0.03**	0.04**	0.01	1.00

* $p < .05$ ** $p < .01$ *** $p < .001$

Table S46. Between-country correlations

Variable	*1*	*2*	*3*	*4*	*5*	*6*	*7*	*8*	*9*	*10*	*11*	*12*	*13*	*14*	*15*	*16*	*17*	*18*
1. Social Mindfulness	1.00																	
2. MES	-0.06	1.00																
3. MES environment	-0.18	0.85***	1.00															
4. Attitude toward immigrants	-0.57***	-0.10	0.13	1.00														
5. Universalism value	-0.12	0.64***	0.64***	-0.12	1.00													
6. Anomie	-0.22	0.26	0.36*	-0.04	0.58***	1.00												
7. Anomie in social fabric	-0.35*	0.28	0.43**	0.14	0.63***	0.93***	1.00											
8. Anomie in leadership	-0.11	0.22	0.28	-0.17	0.49**	0.96***	0.80***	1.00										
9. Benevolence value	-0.12	0.48**	0.42**	-0.28	0.78***	0.39*	0.44**	0.32*	1.00									
10. Care for environment	0.44**	-0.14	-0.36*	-0.36*	-0.29	-0.56***	-0.66***	-0.43**	-0.14	1.00								
11. Tolerance for minorities	0.63***	-0.13	-0.25	-0.67***	0.14	-0.04	-0.19	0.06	0.13	0.37*	1.00							
12. Child value: tolerance/respect	0.27	0.06	-0.02	-0.41*	0.01	-0.15	-0.29	-0.04	0.01	0.61***	0.51**	1.00						
13. Trust	0.33*	-0.34*	-0.52***	-0.11	-0.63***	-0.79***	-0.79***	-0.72***	-0.50**	0.60***	0.13	0.18	1.00					
14. Social capital	0.64***	-0.19	-0.22	-0.11	-0.38*	-0.56***	-0.59***	-0.48**	-0.28	0.58***	0.31	0.25	0.49**	1.00				

15. Collective action	0.49**	-0.29	-0.49**	-0.68***	-0.24	-0.12	-0.32	0.03	-0.04	0.65***	0.62***	0.57***	0.38*	0.34*	1.00			
16. Citizenship	0.49**	-0.18	-0.45**	-0.57***	-0.28	-0.50**	-0.65***	-0.35*	-0.11	0.89***	0.59***	0.64***	0.61***	0.57***	0.85***	1.00		
17. Voice and accountability	0.47**	-0.12	-0.38*	-0.69***	-0.11	-0.09	-0.24	0.03	0.09	0.64***	0.61***	0.52***	0.22	0.32*	0.74***	0.71***	1.00	
18. Child value: Unselfishness	0.17	-0.17	-0.07	-0.30	0.04	0.05	-0.08	0.13	0.05	0.30	0.43**	0.54***	-0.10	-0.02	0.33	0.33	0.21	1.00

* $p < .05$ ** $p < .01$ *** $p < .001$

Supplementary Materials 10: Results for Linear Mixed Model Containing all Country Level Variables for Part 2

Based on advice from reviewers, we placed all nine of our country-level variables from Part 2 in the same model (Table S47). We removed three variables that had an unacceptable Variation Inflation Factor (VIF) above 5 (Table S48). We then conduced a second model (Table S49) with the remaining 6 country-level variables. We found that greater tolerance for minorities as neighbors and greater social capital were associated with social mindfulness. This suggests that many of our constructs may broadly reflect social capital and a tolerance for outgroup members.

Table S47. First Model Examining the Effect of Nine Country-Level Variables on Social Mindfulness

	Social Mindfulness		
Predictors	*Estimates*	*CI*	*p*
(Intercept)	-0.03	-0.34, 0.29	.865
Care for the environment	0.16	-0.63, 0.96	.669
Tolerance for minorities as neighbors	0.84	0.33, 1.36	.003
Child quality: Tolerance/respect	0.02	-0.44, 0.48	.920
Trust	0.45	-0.04, 0.93	.068
Social capital	0.55	0.10, 0.99	.018
Collective action	0.20	-0.54, 0.94	.571
Citizenship Index	-1.08	-2.30, 0.13	.078
Voice and accountability of people	0.16	-0.37, 0.70	.527
Child quality: Unselfishness	-0.04	-0.42, 0.33	.816
**Random Effects**			
Residual	1.00		
Country (intercept)	0.56		
ICC	.36		
N (Country)	29		
Observations	4474		
Marginal R^2 / Conditional R^2	.338 / .576		

Table S48. Variance Inflation Factor for Nine Variables from Table S47.

Variable	VIF
Care for the environment	6.45
Tolerance for minorities as neighbors	3.02
Child quality: Tolerance/respect	2.54
Trust	2.61
Social capital	2.32
Collective action	6.77
Citizenship Index	15.90
Voice and accountability of people	3.09
Child quality: Unselfishness	1.72

Table S49. Second Model Examining the Effect of Six Country-Level Variables with Acceptable VIF on Social Mindfulness

	Social Mindfulness		
Predictors	*Estimates*	*CI*	*p*
(Intercept)	-0.03	-0.31, 0.25	.830
Tolerance for minorities as neighbors	0.53	0.14, 0.92	.010
Child quality: Tolerance/respect	-0.10	-0.48, 0.29	.608
Trust	0.05	-0.28, 0.37	.772
Social capital	0.49	0.14, 0.83	.007
Voice and accountability of people	0.07	-0.31, 0.44	.721
Child quality: Unselfishness	0.05	-0.31, 0.40	.787
**Random Effects**			
Residual	1.00		
Country (intercept)	0.61		
ICC	.38		
N (Country)	34		
Observations	5246		
Marginal R^2 / Conditional R^2	.297 / .562		

References

1. Van Doesum, N. J., Van Lange, D. A. W. & Van Lange, P. A. M. Social mindfulness: Skill and will to navigate the social world. *J. Pers. Soc. Psychol.* **105**, 86–103 (2013).
2. Van Lange, P. A. M., Vinkhuyzen, A. A. E. & Posthuma, D. Genetic influences are virtually absent for trust. *PLoS One* **9**, e93880 (2014).
3. Goodman, E. *et al.* Adolescents' perceptions of social status: Development and evaluation of a new indicator. *Pediatrics* **108**, 1–8 (2001).
4. Inglehart, R. *et al. World Values Survey: Round Six - Country-Pooled Datafile*. Madrid, Spain & Vienna, Austria: JD Systems Institute & WVSA Secretariat. (2018) doi:doi.org/10.14281/18241.7.
5. EVS. *European Values Study: Integrated Dataset (EVS2017)*. GESIS Data Archive, Cologne. ZA7500 Data file Version 5.0.0 (2017) doi:10.4232/1.13560.
6. The World Bank. Worldwide Governance Indicators. (2019).
7. Economist Intelligence Unit. Democracy Index 2019: A year of democratic setbacks and popular protest. *The Economist* https://www.eiu.com/n/campaigns/democracy-index-2020/ (2019).
8. Schwab, K. *The Global Competitiveness Report*. (2019).
9. Reporters Without Borders. World Press Freedom Index. https://rsf.org/en/index (2019).
10. Wendling, Z. A., Emerson, J. W., de Sherbinin, A. & Esty, D. C., et al. Environmental Performance Index. https://epi.yale.edu (2020).
11. Hofstede Insights. Compare Countries. https://www.hofstede-insights.com/product/compare-countries/ (2020).
12. The World Bank. GNI per capita, Atlas method (current US$). https://data.worldbank.org/indicator/NY.GNP.PCAP.CD (2019).
13. The World Bank. GDP per capita (current US$). *The World Bank* https://data.worldbank.org/indicator/NY.GDP.PCAP.CD (2020).
14. World Factbook. Distribution of family income - Gini index. *World Factbook* https://www.cia.gov/the-world-factbook/field/gini-index-coefficient-distribution-of-family-income/country-comparison (2018).
15. Crimston, C. R., Bain, P. G., Hornsey, M. J. & Bastian, B. Moral expansiveness: Examining variability in the extension of the moral world. *J. Pers. Soc. Psychol.* **111**, 636–653 (2016).
16. Jetten, J., Mols, F. & Postmes, T. Relative deprivation and relative wealth enhances anti-immigrant sentiments: The V curve re-examined. *PLoS One* **10**, 1–24 (2015).
17. Schwartz, S. H. An overview of the Schwartz theory of basic values. *Online Readings Psychol. Cult.* **2**, 2307–0919 (2012).
18. Teymoori, A. *et al.* Revisiting the measurement of anomie. *PLoS One* **11**, 1–27 (2016).
19. Sprong, S. *et al.* "Our country needs a strong leader right now": Economic inequality enhances the wish for a strong leader. *Psychol. Sci.* **30**, 1625–1637 (2019).
20. US News. Citizenship. https://www.usnews.com/news/best-countries/citizenship-rankings (2019).

21. Ortiz-Ospina, E. & Roser, M. Trust. *Our World In Data* https://ourworldindata.org/trust (2016).
22. Global Sustainable Competitiveness Index. Social Capital Index: World Map. https://solability.com/the-global-sustainable-competitiveness-index/the-index/social-capital (2020).
23. Kaufmann, D. & Kraay, A. Worldwide Governance Indicators. http://info.worldbank.org/governance/wgi/ (2019).
